# Supplementary material for: Ena/VASP Proteins Cooperate with the WAVE Complex to Regulate the Actin Cytoskeleton
Source: Dev Cell. 2014 Sep 8;30(5):569–84. doi: 10.1016/j.devcel.2014.08.001 (PMC4165403; doi:10.1016/j.devcel.2014.08.001)
Supplement: Document S1. Figures S1–S3 [file mmc1.pdf]

Developmental Cell, Volume *30*

Supplemental Information

Ena/VASP Proteins Cooperate with the WAVE Complex to Regulate the Actin Cytoskeleton

Xing Judy Chen, Anna Julia Squarr, Raiko Stephan, Baoyu Chen, Theresa E. Higgins, David J. Barry, Morag C. Martin, Michael K. Rosen, Sven Bogdan, and Michael Way

Supplemental figure legends

Figure S1: The interaction between the EVH1 domain of Ena/VASP proteins and Abi promotes cell migration, related to figure 2. **(A)** Far western analysis with GST or the GST-EVH1 domains of Mena, VASP and Evl on arrays of overlapping peptides covering residues 320-415 of human Abi1. Similar peptides were found to interact with all three EVH1 domains. Red arrowheads indicate the examples of non-identical peptide binding patterns. Green arrows correspond to the first and last peptides listed in (B). **(B)** Shows a list and relative binding of human Abi1 peptides to the EVH1 domains of Mena, VASP and Evl. **(C)** Immunoblot analysis of Abi1 in HT1080 cells stably expressing the indicated GFP tagged protein treated with control (Ctrl) or siRNA against the 3' UTR of Abi1 message (Abi). Long and short exposures with anti-GFP reveal that GFP is greatly over expressed compared to GFP-Abi1. Anti-Abi1, however, reveals that the GFP-Abi1 proteins are expressed at comparable levels to endogenous Abi1 (see GFP Ctrl lane). **(D)** Phase images of HT1080 cells treated with the indicated siRNA and expressing GFP, GFP-Abi or Abi1ΔEVH1 migrating into a scratch at the indicated time. The yellow lines correspond to the migration front and the scale bar = 300μm.

Figure S2: Purified WRC and VASP proteins used for in vitro actin polymerization assays, related to figure 3. **(A)** Coomassie stained SDS-PAGE gel showing purified recombinant proteins used in actin polymerization assays. **(B)** In *vitro* pull-down assays with recombinant proteins reveals that the Mena EVH1 domain interacts directly with WAVE1 and WAVE2 but not WAVE3.

Figure S3: Overexpression of the Abi variants in a wild type background does not lead to dominant negative effects on axon targetting, related to figure 7.
(A) Representative images of projection patterns of all photoreceptor axons of the indicated genotypes. Scale bar = 15μm **(B)** Quantification of the frequency of optic lobes with axonal bundles and the number of gaps per optic lobe of the indicated genotypes. * indicates $p < 0.05$ (ANOVA).

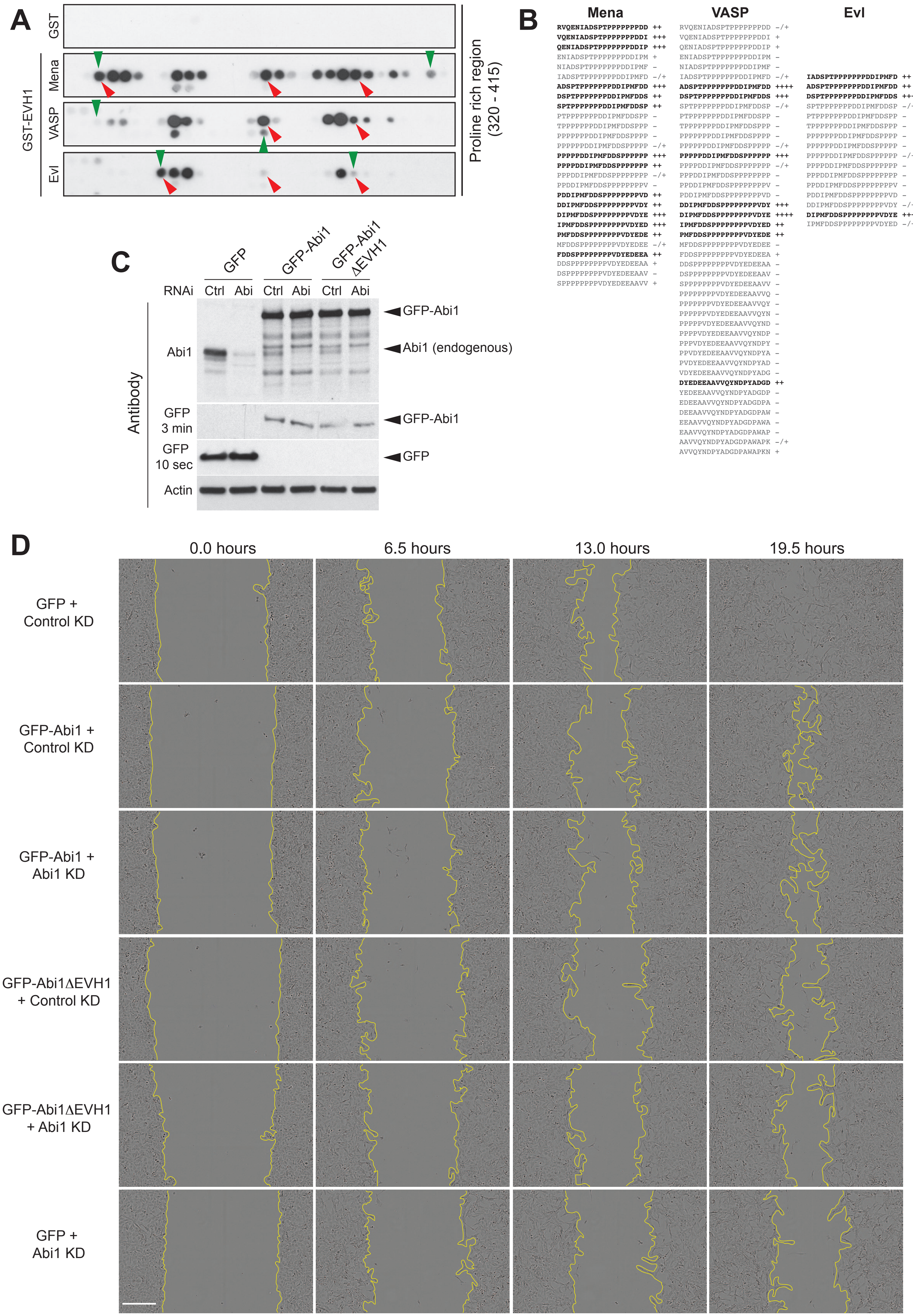

Figure S1 Chen et al 2013

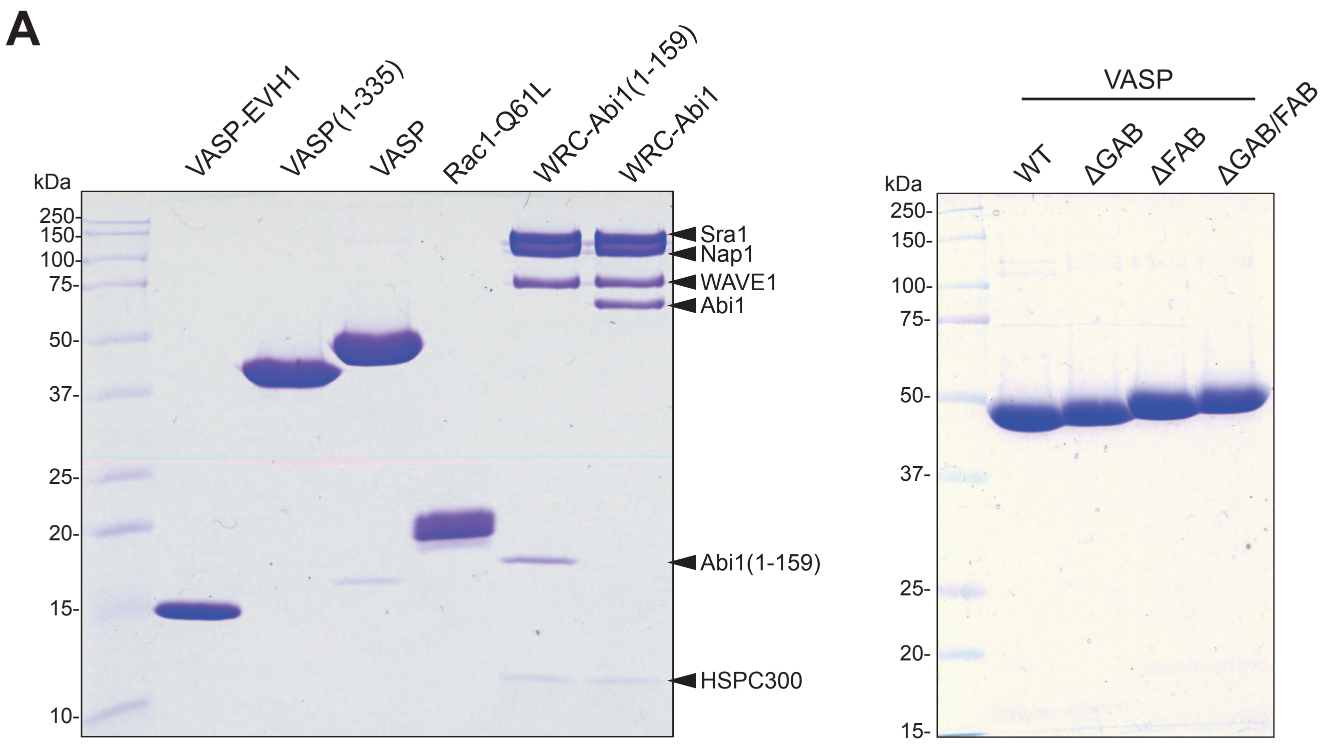

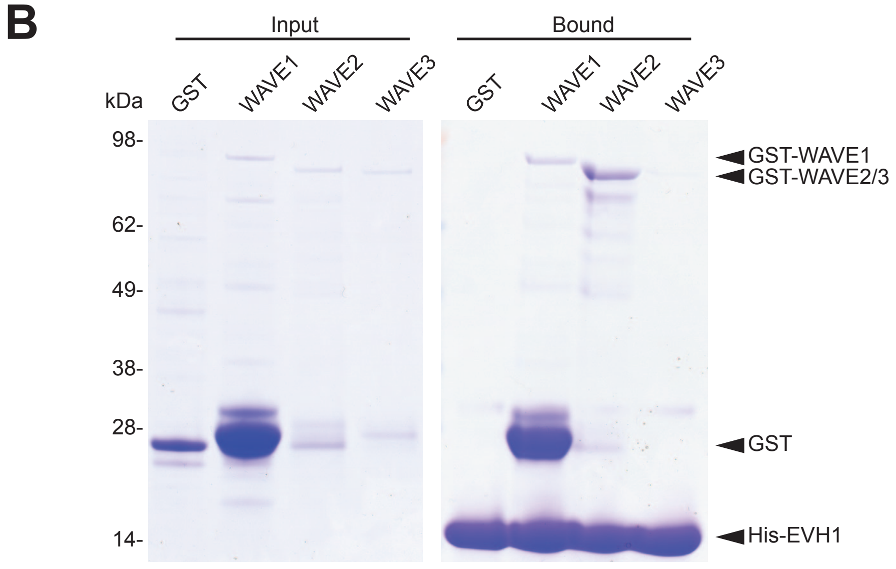

Figure S2 Chen et al 2013

A

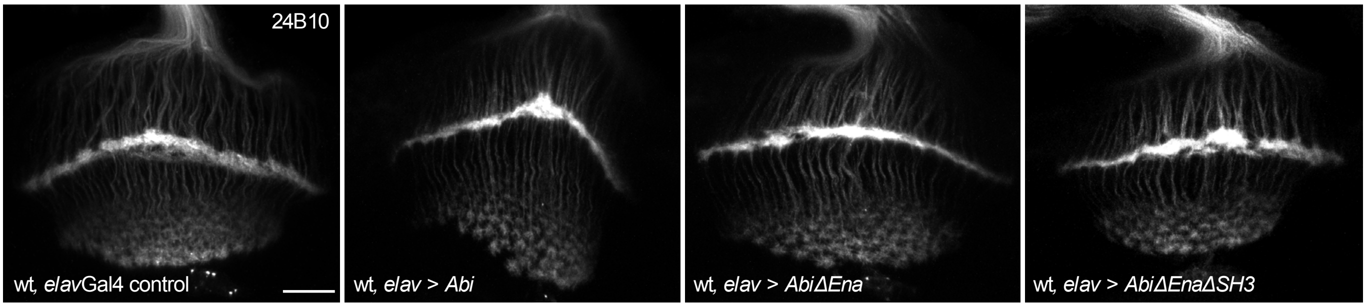

B

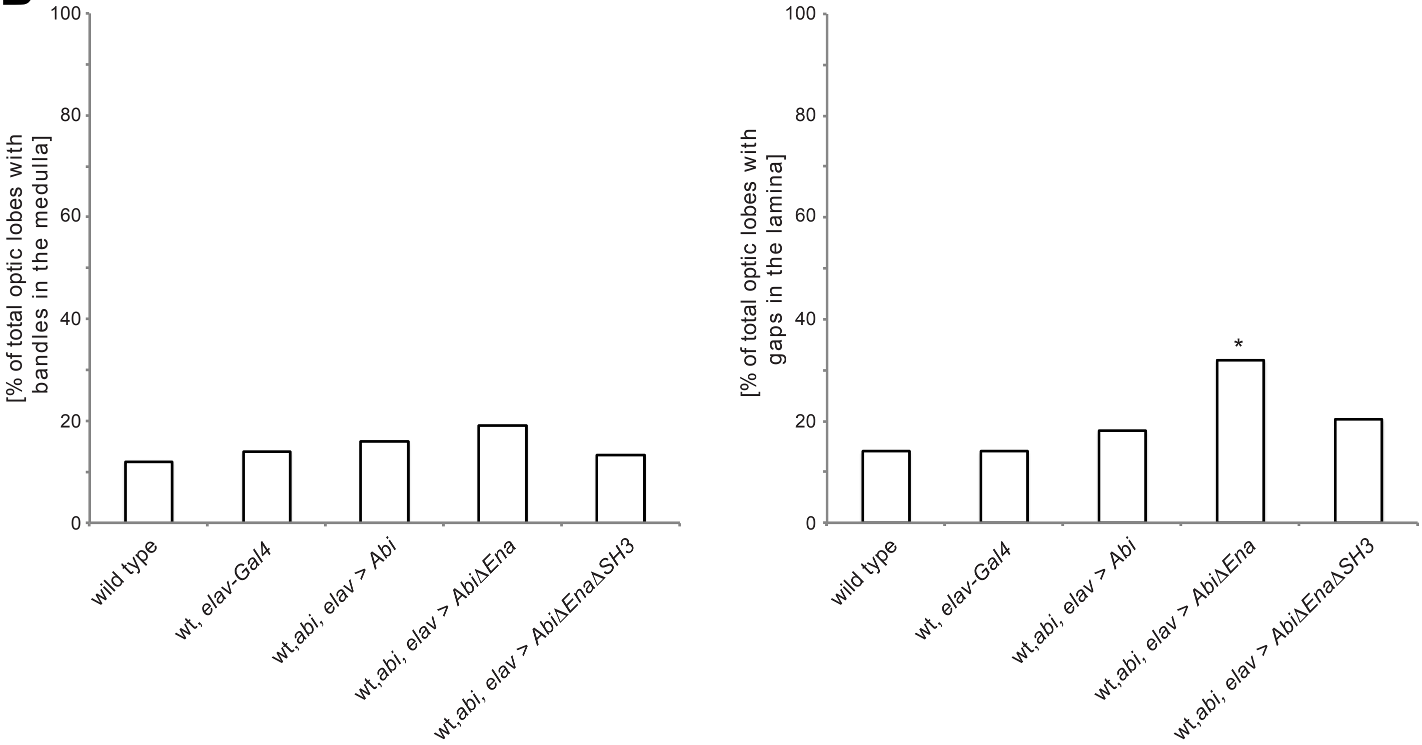

Figure S3 Chen et al 2013

Supplemental Movie legends

Movie S1: Representative movie of larval Abi mutant macrophages expressing dAbi and dAbiΔEna, related to figure 6. The segmented cell boundaries are color-coded to represent the local membrane velocity – green depicts membrane expansion and red retraction. The time is shown in minutes and seconds.
