# Supplementary material for: Ena/VASP Proteins Cooperate with the WAVE Complex to Regulate the Actin Cytoskeleton
Source: Dev Cell. 2014 Sep 8;30(5):569–84. doi: 10.1016/j.devcel.2014.08.001 (PMC4165403; doi:10.1016/j.devcel.2014.08.001)
Supplement: Document S2. Article plus Supplemental Information [file mmc3.pdf]

Developmental Cell
Article

Ena/VASP Proteins Cooperate with the WAVE Complex to Regulate the Actin Cytoskeleton

Xing Judy Chen,[1,5] Anna Julia Squarr,[2,4] Raiko Stephan,[2,4,6] Baoyu Chen,[3,4] Theresa E. Higgins,[1] David J. Barry,[1] Morag C. Martin,[1,7] Michael K. Rosen,[3] Sven Bogdan,[2,*] and Michael Way[1,*]
[1]Cell Motility Laboratory, London Research Institute, Cancer Research UK, 44 Lincoln's Inn Fields, London WC2A 3LY, UK
[2]Institute of Neurobiology, University of Muenster, Badestrasse 9, 48149 Muenster, Germany
[3]Howard Hughes Medical Institute and Department of Biophysics, UT Southwestern Medical Center, Dallas, TX 75390, USA
[4]Co-second author
[5]Present address: Randall Division of Cell and Molecular Biophysics, King's College London, London SE1 1UL, UK
[6]Present address: Friedrich Miescher Institute, Maulbeerstrasse 66, 4058 Basel, Switzerland
[7]Present address: College of Life Science, University of Dundee, Dundee DD1 5EH, UK
*Correspondence: sbogdan@uni-muenster.de (S.B.), michael.way@cancer.org.uk (M.W.)
http://dx.doi.org/10.1016/j.devcel.2014.08.001
This is an open access article under the CC BY license (http://creativecommons.org/licenses/by/3.0/).

SUMMARY

Ena/VASP proteins and the WAVE regulatory complex (WRC) regulate cell motility by virtue of their ability to independently promote actin polymerization. We demonstrate that Ena/VASP and the WRC control actin polymerization in a cooperative manner through the interaction of the Ena/VASP EVH1 domain with an extended proline rich motif in Abi. This interaction increases cell migration and enables VASP to cooperatively enhance WRC stimulation of Arp2/3 complex-mediated actin assembly in vitro in the presence of Rac. Loss of this interaction in *Drosophila* macrophages results in defects in lamellipodia formation, cell spreading, and redistribution of Ena to the tips of filopodia-like extensions. Rescue experiments of *abi* mutants also reveals a physiological requirement for the Abi:Ena interaction in photoreceptor axon targeting and oogenesis. Our data demonstrate that the activities of Ena/VASP and the WRC are intimately linked to ensure optimal control of actin polymerization during cell migration and development.

INTRODUCTION

Ena/VASP proteins regulate cell migration by promoting actin polymerization at the plasma membrane via antagonizing actin filament capping and acting as processive actin polymerases (Barzik et al., 2005; Bear et al., 2002; Breitsprecher et al., 2008, 2011; Hansen and Mullins, 2010; Pasic et al., 2008). Each family member consists of an N-terminal EVH1 domain, a central proline-rich region, and a C-terminal EVH2 domain (Bear and Gertler, 2009). The EVH2 domain, which contains monomeric and F-actin binding sites, is responsible for promoting actin polymerization (Barzik et al., 2005; Breitsprecher et al., 2008, 2011; Pasic et al., 2008). In contrast, the EVH1 domain mediates intracellular targeting of Ena/VASP proteins by interacting with a sequence (D/E)FPPPPX(D/E)(D/E), which is referred to as the "FPPPP" motif because these residues are essential for binding (Bear et al., 2000; Niebuhr et al., 1997; Peterson and Volkman, 2009). Ena/VASP proteins are recruited to focal adhesions by zyxin, which contains four "FPPPP" motifs (Drees et al., 2000; Garvalov et al., 2003; Hoffman et al., 2006). The ability of Ena/VASP proteins to control cell migration, however, depends on their recruitment to the leading edge (Bear et al., 2000, 2002), by "FPPPP" motif containing MRL proteins (Mig10, RIAM, and Lamellipodin; Coló et al., 2012; Jenzora et al., 2005; Krause et al., 2004; Lafuente et al., 2004; Quinn et al., 2006).

Of all the proteins interacting with the EVH1 domain of Ena/VASP proteins, Tes, a focal adhesion protein, stands out as the only one that lacks an "FPPPP" motif (Coutts et al., 2003; Garvalov et al., 2003). Tes negatively regulates the localization of Mena at focal adhesions and also inhibits Mena-dependent cell migration (Boëda et al., 2007). Tes interacts with Mena via its C-terminal LIM3 domain and is unique in being the only protein that binds a single Ena/VASP family member (Boëda et al., 2007, 2011; Garvalov et al., 2003). Given the interaction of Tes with Mena, we sought to identify additional atypical EVH1 binding partners that also lack "FPPPP" motifs. We found that the EVH1 domain interacts directly with Abi, a component of the WAVE regulatory complex (WRC), which plays an essential role in driving cell migration by activating the Arp2/3 complex in response to Rac signaling (Bisi et al., 2013). Our observations demonstrate that the EVH1:Abi interaction enhances cell migration and the ability of Rac-activated WRC to promote Arp2/3-mediated actin polymerization as well as the function of WRC in vivo in *Drosophila*.

RESULTS

The EVH1 Domain of Ena/VASP Proteins Binds Directly to Abi in the WAVE Complex

To identify Ena/VASP binding proteins lacking "FPPPP" motifs we performed pull-down assays with GST-tagged EVH1 domain of Mena on lysates from MV^{D7} cells, which lack endogenous

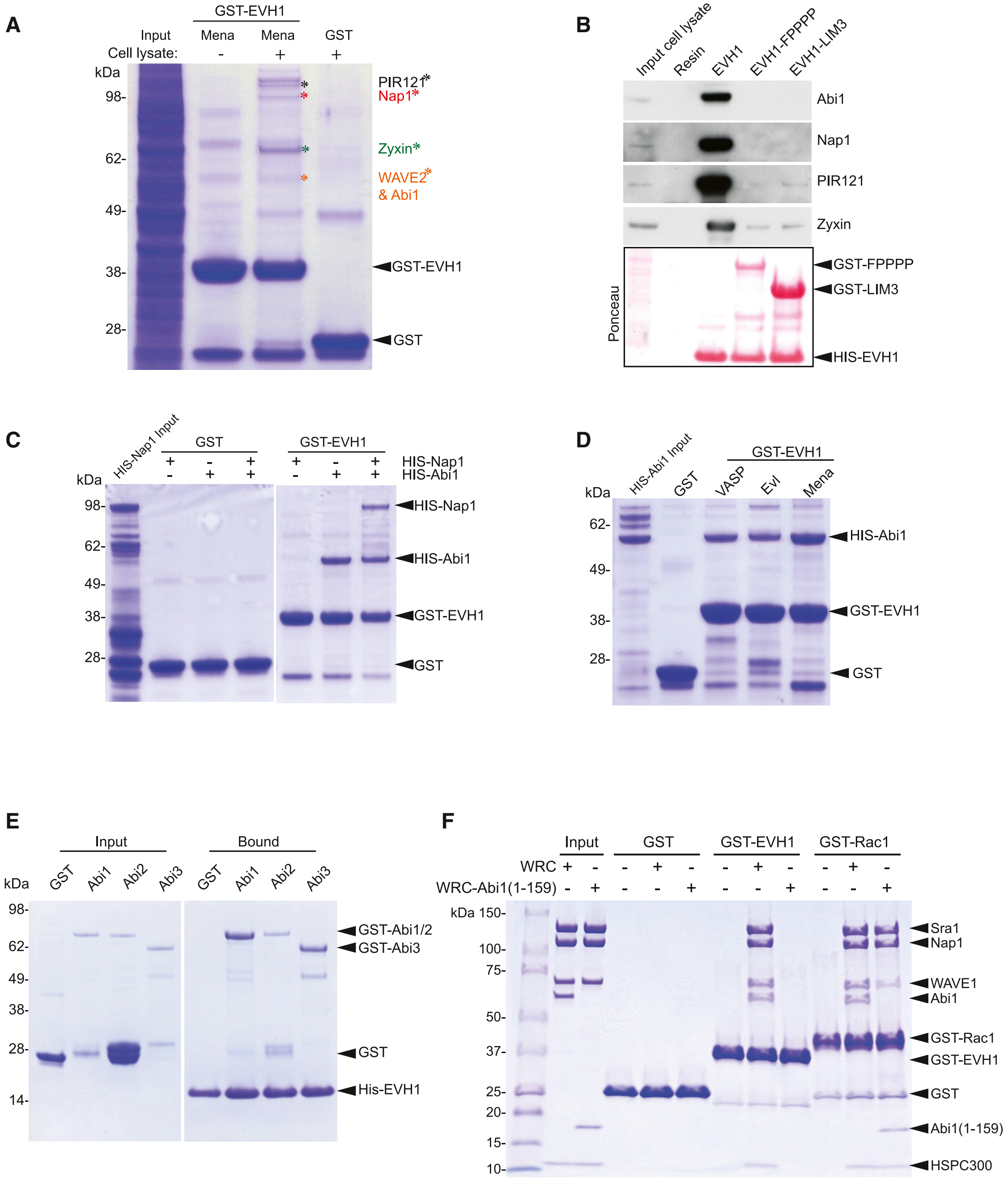

Figure 1. The Ena/VASP EVH1 Domain Interacts Directly with Human Abi1

(A) Mass spectrometric analysis reveals that GST-EVH1 Mena, but not GST, retains Abi1, Nap1, PIR121, WAVE2, and zyxin from MV^{D7} cell lysates.

(B) Immunoblot analysis reveals that the FPPPP domain of zyxin or the Tes LIM3 domain inhibits the interaction of His-EVH1 Mena with the WAVE complex or zyxin. The ponceau stain shows the proteins on the resins.

(C) Pull-down assays with recombinant proteins reveals that the Mena EVH1 domain interacts directly with Abi1 but not Nap1.

(D) The EVH1 domains of VASP, Evl, and Mena interact with Abi1.

(E) The EVH1 domain of Mena can interact with Abi1, 2 and 3.

(F) In vitro pull-down assays reveal that in contrast to Rac1, the EVH1 domain of VASP can only bind WRC and not WRC-Abi1(1–159).

Mena and VASP (Figure 1A). Mass spectrometry analysis of the resulting bands identified zyxin and four subunits of the WRC: Abi1, Nap1, PIR121, and WAVE2 (Figure 1A). Western blot analysis confirmed that the Mena EVH1 domain interacts with the WRC (Figure 1B). Furthermore, this interaction depends on its FPPPP binding activity, because preincubation of the resin with the FPPPP region of zyxin (EVH1-FPPPP) or the LIM3 domain of Tes (EVH1-LIM3) inhibited binding (Figure 1B). Using

bacterially expressed proteins, we found that the EVH1 domains of Mena, Evl, and VASP are all capable of interacting directly with Abi1 (Figures 1C and 1D). The EVH1 domain of Mena can also bind Abi2 and Abi3 (Figure 1E). Human and mouse Abi1 lack "FPPPP" motifs, but do contain extensive proline rich regions that may contain alternative EVH1 binding sites. Consistent with this, pull-down assays reveal that a WAVE complex containing Abi1(1-159) lacking its C-terminal proline rich region and SH3 domain can bind Rac1 but not the VASP EVH1 domain (Figure 1F).

The EVH1 Domain Interacts with a Non-"FPPPP" Motif in Human Abi

The "FPPPP" EVH1 binding motif was first identified in ActA by screening a series of overlapping peptides using a far western approach (Niebuhr et al., 1997). Using the same method, we found that the Mena EVH1 domain interacts with a series of peptides covering residues 352–394 in the proline rich region of human Abi1 (Figure 2A). The EVH1 domains of Evl and VASP also bound the same region although the peptide binding patterns were not completely identical (Figures S1A and S1B available online). Probing three overlapping peptide arrays containing systematic amino acid substitutions at each position we found that mutation of proline residues 366–368 (peptide 1) and 383–385 (peptide 3) as well as phenylalanine 375 (peptide 2) significantly reduced binding (Figure 2B). Substitution of prolines 366–368 and 383–385 with glycine as well as phenylalanine 375 to alanine (termed AbiΔEVH1) abrogated all peptide interactions with the Mena EVH1 domain (Figure 2C). Pull-down assays on lysates from cells expressing GFP-AbiΔEVH1 confirmed that these mutations disrupt the interaction of Abi1 with Mena but not the rest of the WAVE complex (represented by the PIR121 subunit; Figure 2D). To investigate the impact of the loss of the interaction between Abi1 and Ena/VASP proteins on cell migration, we stably expressed GFP-tagged Abi or AbiΔEVH1 in HT1080 cells (Figure S1C). Both GFP-tagged proteins are recruited to the leading edge of migrating cells (Figure 2E). Expression of AbiΔEVH1 but not Abi, however, retarded the migration of HT1080 cells into a scratch (Figures 2F and S1D). Conversely, in the absence of endogenous Abi1, GFP-tagged AbiΔEVH1 was less effective at promoting cell migration than the wild-type protein (Figures 2F and S1D).

VASP Enhances WRC Activity in the Presence of Rac

Given the impact of AbiΔEVH1 on cell migration, we wondered whether the interaction of EVH1 with Abi1 modulates the activity of VASP and/or the WRC. To explore this possibility, we performed in vitro actin polymerization assays using recombinant VASP and WRC (Figures 3 and S2). In the absence of its canonical activator, Rac1, the WRC cannot stimulate Arp2/3-mediated actin assembly (Figure 3A, red solid curve). In contrast, VASP promotes actin assembly (Figure 3A, black dotted curve). Further addition of the WRC produced no further changes in actin assembly (Figure 3A, red dotted curve), indicating that VASP does not activate the inhibited state of the WRC, or vice versa.

In our assay conditions (100 mM KCl), the WAVE1 VCA peptide only slightly increases actin assembly by the Arp2/3 complex (Figure 3B, green solid curve). Saturating concentrations of Rac1 increase WRC-mediated actin assembly to this same level (Figure 3B, red solid curve). Addition of VASP to the Rac1-activated WRC significantly increases the extent of actin polymerization (Figure 3B red dotted curve) in a dose-dependent manner (Figure 3C). This increase is not the result of simple summation of the activity of VASP and the Arp2/3 complex, as when the activated WRC is substituted with the VCA peptide, the increase in actin polymerization is only additive (Figure 3B, green curves). To examine whether direct interaction between VASP and the WRC is responsible for the enhancement in actin assembly, we tested a variant of the WRC (WRC-Abi1[1–159]), which cannot bind the EVH1 domain as it lacks the proline rich region of Abi1 (Figure 1F). WRC-Abi1(1–159) can be activated by Rac1, but failed to recapitulate the increased activity with VASP seen for the full-length WRC (Figure 3B, compare blue and red dotted curves). Thus, the increased activity requires a direct interaction between VASP and the WRC.

We next sought to understand how the VASP-WRC interaction promotes enhanced actin assembly. In principle, this could arise from increased activity of either VASP or the WRC. When the Arp2/3 complex is removed from assays containing both VASP and activated WRC, the total activity dropped to the level of VASP alone (Figure 3B, orange curve). Therefore, the increased activity depends on the Arp2/3 complex, suggesting that VASP increases the activity of WRC, but not vice versa. Because the isolated EVH1 domain of VASP cannot increase the activity of the WRC (Figure 3D), we sought to discover which other regions of VASP are required for this effect. In addition to the EVH1 domain, VASP has a C-terminal EVH2 domain, which includes a G-actin binding motif (GAB), an F-actin binding motif (FAB), and a coiled coil maintaining VASP as a constitutive tetramer (Bear and Gertler, 2009). Deletion of the coiled coil, which generates a monomeric form of VASP (VASP [1–335]), fails to recapitulate the increased activity seen with the full-length VASP (Figure 3D). This suggests that tetramerization of VASP is required for its activity toward the WRC. Furthermore, the FAB motif is also necessary, since its mutation, which completely disables VASP in actin assembly (Breitsprecher et al., 2008, 2011; Hansen and Mullins, 2010; Pasic et al., 2008), no longer allows VASP to enhance actin assembly by the WRC even at a concentration of 5 μM (Figure 3E). In contrast, mutation of the GAB motif alone, which significantly decreases the activity of VASP in actin polymerization, still allows VASP to stimulate WRC activity (but only at high concentrations of 1–5 μM range; Figure 3F). Taken together, our data suggest that disrupting the actin polymerization activity of VASP impairs its ability to enhance WRC activity.

The Ena:Abi Interaction Is Evolutionarily Conserved

To facilitate further analysis of the physiological role of the interaction between Ena/VASP proteins and the WRC, we switched to *Drosophila* because it only has a single isoform for each protein. *Drosophila* Abi (dAbi) and the EVH1 domain of Ena have 38% and 72% sequence identity to their respective human counterparts (Figure 4A). *Drosophila* Abi also lacks the EVH1 binding motif we identified in human Abi1 (Figure 4A). Nevertheless, the EVH1 domain of Ena still retains the WAVE complex from *Drosophila* S2 cell lysates (Figure 4B). Using a far western approach, we found that the EVH1 domain of Ena bound two sets of dAbi peptides containing an "LPPPP" motif at positions 311–315 and 374–378 (Figures 4C and D). Ena/VASP EVH1

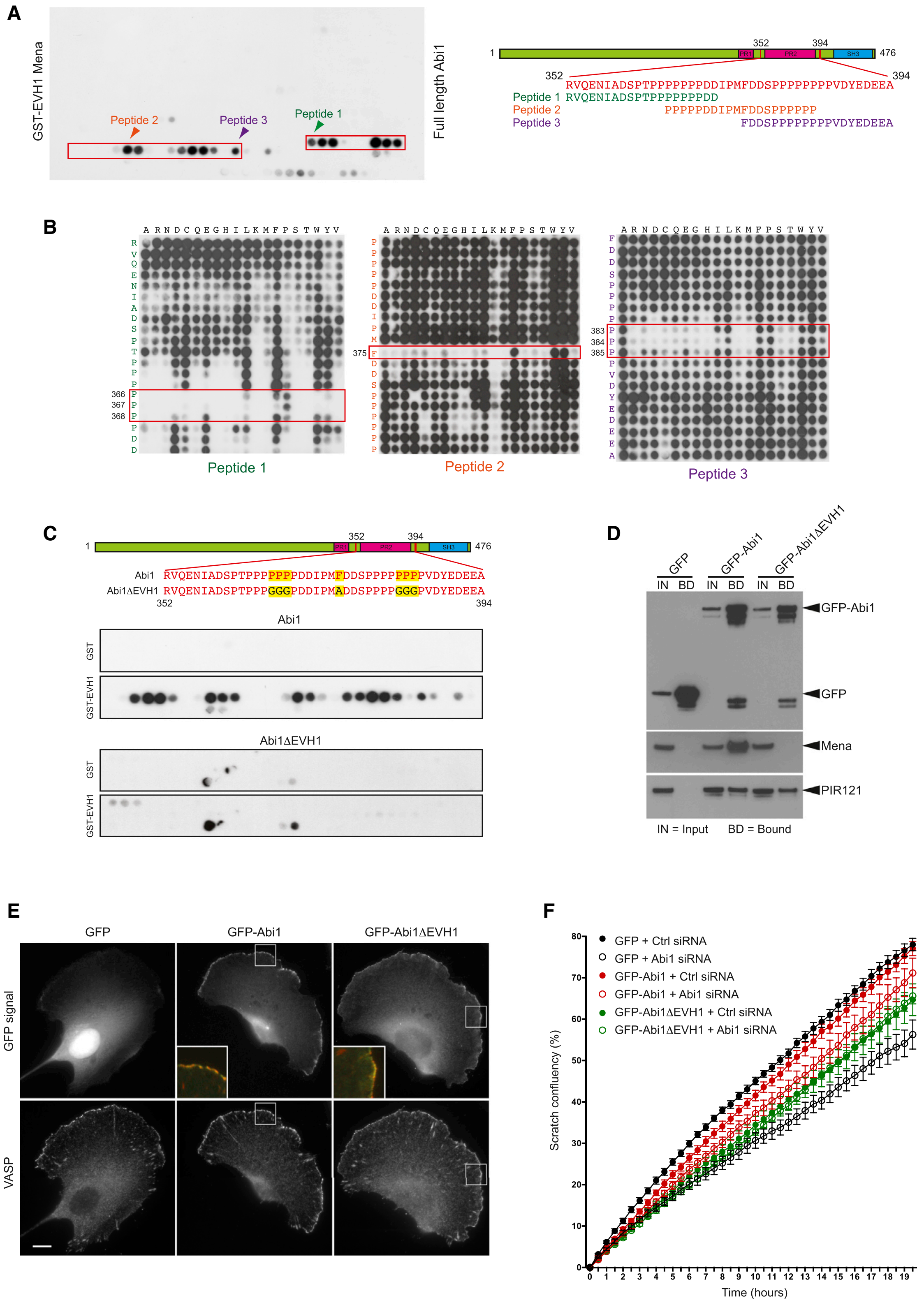

Figure 2. Identification of the EVH1 Binding Site in Human Abi1

(A) Far western analysis reveals that GST-tagged EVH1 domain of Mena interacts with peptides corresponding to residues 352–394 in the proline rich region of Abi1 (red box). Arrowheads indicate overlapping peptides selected for systematic mutagenesis.

(legend continued on next page)

domains can interact with “LPPPP” peptides, albeit with reduced affinity compared to “FPPPP” motifs (Ball et al., 2000; Niebuhr et al., 1997; Peterson and Volkman, 2009). Far western analysis of peptide arrays containing systematic substitutions at each position confirmed that both LPPPP motifs are important for EVH1 binding (Figure 4E).

To establish if these motifs are important in the context of dAbi, we performed pull-down assays on wild-type and mutants in which the two “LPPPP” motifs were mutated to AGGGG, either alone or in combination (Figure 5A). Substitution of residues 311–315 (Mut1) substantially weakened but did not completely abolish the interaction of dAbi with the Ena EVH1 domain. In contrast, mutation of residues 374–378 (Mut2) resulted in a loss of binding (Figure 5A). Mutation of both “LPPPP” motifs did not, however, disrupt the ability of dAbi (dAbiΔEna) to colocalize with WAVE at the plasma membrane of *Drosophila* S2 cells (Figure 5B). The dAbiΔEna mutant was also as effective as the wild-type protein at rescuing the “spiky” morphology of *Drosophila* S2 cells induced by RNAi-mediated loss of endogenous dAbi (Figure 5C; Kunda et al., 2003; Rogers et al., 2003). To examine whether the interaction between Ena and dAbi helps to stabilize the WRC at the plasma membrane, we performed fluorescence recovery after photobleaching (FRAP) experiments on *Drosophila* S2 cells treated with RNAi targeting the 3′ UTR of endogenous dAbi and expressing GFP-tagged Abi or AbiΔEna. We found that the loss of Ena binding results in a statistically significant ~1.38-fold increase in the exchange rate of dAbi at the plasma membrane (Figure 5D). Importantly, pull-down assays on S2 lysates demonstrate that the loss of the interaction with Ena did not disrupt the ability of dAbi to incorporate into the WAVE complex (Figure 5E).

Drosophila Macrophage Lamellipodia Formation Depends on the Ena:Abi Interaction

To investigate the physiological significance of the interaction between Ena and Abi in regulating WRC functions in vivo, we took advantage of *abi* mutant flies (Stephan et al., 2011). We first examined the consequences of the loss of the Abi:Ena interaction in *Drosophila* macrophages. Structured-illumination microscopy analysis of spreading wild-type macrophages reveals a highly polarized actin cytoskeleton with a broad lamellipodial cell front (Figures 6A and 6B). Macrophage-specific knockdown of WAVE in larval macrophages using the *hemolectin*-Gal4 driver completely disrupts lamellipodia formation (Figure 6A; Sander et al., 2013). The *abi* mutant macrophages also had a similar “spiky” morphology (Figures 6A and 6B). These defects in cell morphology were substantially rescued by ubiquitous reexpression of wild-type Abi but not by the mutant lacking both Ena binding motifs (AbiΔEna) from the same genomic locus (Figures 6A and 6B). To further analyze differences in lamellipodia protrusions and cell shape of rescued macrophages, we performed live cell imaging of larval macrophages expressing cytoplasmic GFP. We found that cells expressing Abi exhibit more stable and periodic membrane protrusions, whereas the AbiΔEna population are significantly less circular (more spiky) and have a reduced rate of membrane protrusion, (Figure 6C; Movie S1). Furthermore, in contrast to wild-type macrophages, Ena is no longer at the leading edge of lamellipodial protrusions but becomes relocalized to the tips of filopodia-like protrusions and along stress fiber-like actin bundles when it cannot interact with Abi (Figure 6D).

Drosophila Photoreceptor Targeting and Oogenesis Requires Ena:Abi Interactions

In vivo, Abi and a functional WRC are required in the *Drosophila* larval visual system for the correct axonal targeting of photoreceptor neurons (R-cells) to their respective optic ganglions in the fly brain (Stephan et al., 2011). Remarkably, we found that the loss of the ability of Abi to interact with Ena resulted in a similar defect in R-cell targeting as the complete loss of Abi (Figures 7A and 7B). We also examined the impact of an Abi mutant lacking its Ena binding sites and the C-terminal SH3 domain (AbiΔEnaΔSH3) because an Abi transgene lacking its C-terminal SH3 domain is able to substantially rescue abi mutant phenotypes (Stephan et al., 2011). In contrast to AbiΔEna, the expression of AbiΔEnaΔSH3 surprisingly rescued the *abi*-dependent R-cell targeting defects (Figures 7A and 7B). Overexpression of the Abi variants in a wild-type background, however, did not affect R-cell targeting excluding any dominant effects (Figure S3).

Next, we analyzed the cellular requirement for the Abi:Ena interaction during oogenesis because, in contrast to the visual system, WRC and Ena control cell-autonomous actin-based structures that are essential for normal egg development (Gates et al., 2009; Zallen et al., 2002). Loss of *wave* and *arp2/3* in the germline results in small and abnormally shaped eggs (Hudson and Cooley, 2002; Zallen et al., 2002). A similar dumpless mutant phenotype is also observed in flies lacking *abi* in the germline (Zobel and Bogdan, 2013). Rescue experiments using a pUASt-Abi transgene that is only expressed in somatic follicle cells and not the germline rescues the lethality of *abi* mutants (Zobel and Bogdan, 2013). However, the integrity of cortical actin in nurse cells within the egg chamber is disrupted and the rescued female flies are completely sterile (Figures 7C and 7D). To overcome this problem, we generated pUASp transgenes for efficient germline expression (Rørth, 1998) and performed rescue experiments with the different Abi variants.

(B) Far western analysis of the three peptides shown in (A) containing systematic single amino acid substitutions at each position. The residues that are most essential for EVH1 binding are highlighted in red boxes.

(C) Far western analysis on peptides corresponding to residues 320–415 of human Abi1 reveals that mutation of the residues identified in B (highlighted in yellow) abrogates the interaction with the GST-EVH1 domain of Mena.

(D) Immunoprecipitations reveal that whereas GFP-tagged Abi1 and Abi1ΔEVH1 incorporate into the WAVE complex (represented by PIR121), only Abi1 can interact with Mena.

(E) Representative images showing the localization of GFP, GFP-Abi, or Abi1ΔEVH1 together with VASP in HT1080 cells. The inserts highlight the colocalization of GFP-Abi and Abi1ΔEVH1 (green) with VASP (red) at the leading edge. Scale bar represents 10 μm.

(F) The graph shows the rate at which HT1080 cells treated with the indicated siRNA and expressing GFP, GFP-Abi, or Abi1ΔEVH1 migrate into a scratch. The graph is derived from six replicates and the error bars represent the SEM.

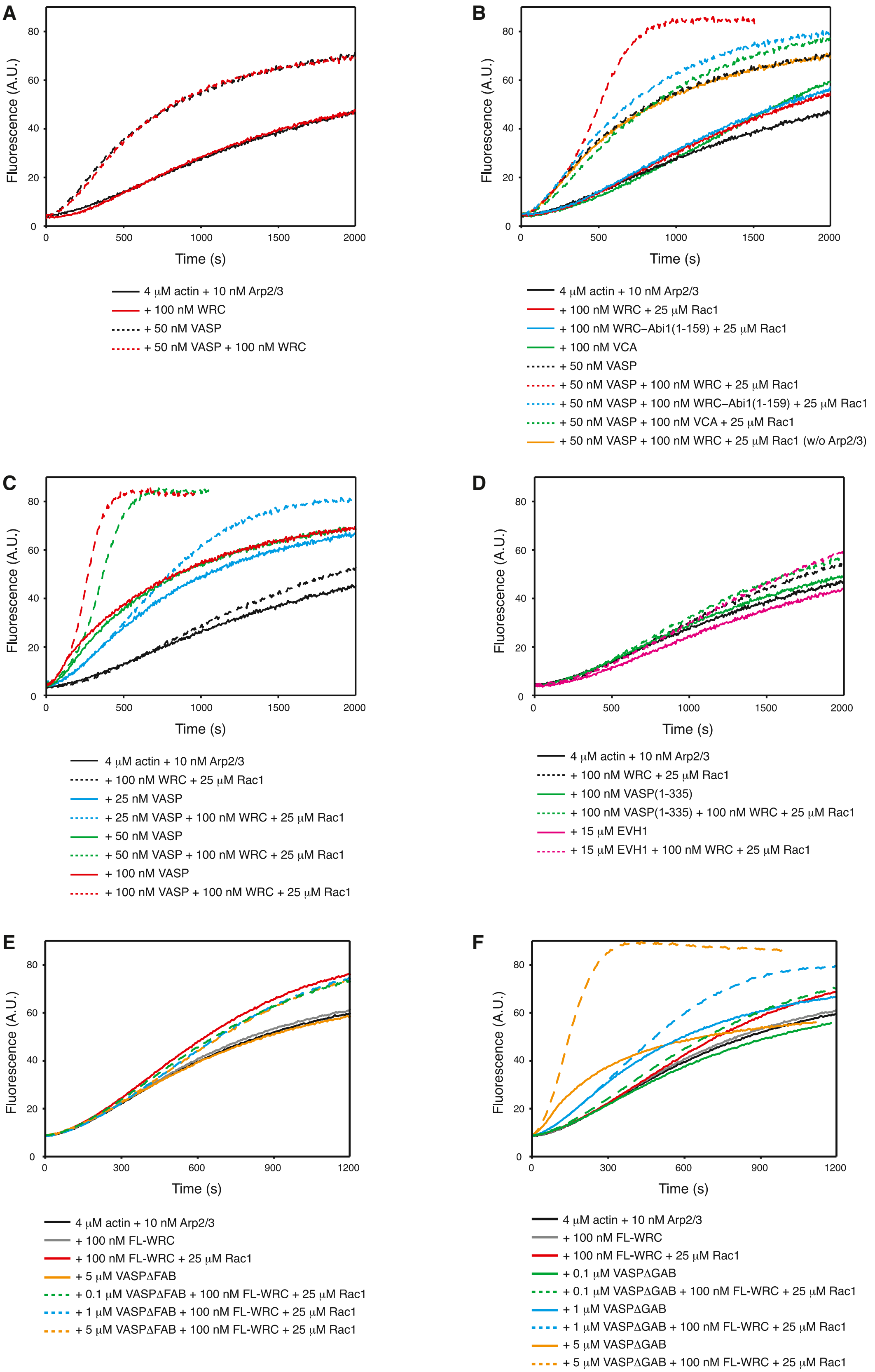

(legend on next page)

Reexpression of wild-type Abi rescues the egg morphology defects and female sterility of *abi* mutant flies (Figures 7C and 7D). In contrast, *abi* mutant flies expressing AbiΔEna are completely sterile, containing smaller and abnormally round eggs (Figures 7C–7E). This round-egg phenotype resembles those seen in *rac1*, *rac2* mutant follicle cell clones defective for egg chamber elongation rather than flies lacking *abi* in the germline (Conder et al., 2007; Zobel and Bogdan, 2013). Supporting this notion, we observed a similar round-egg phenotype when we suppress WRC function in follicle cells using an sra-1 RNAi transgene (Figure 7F).

Late-stage AbiΔEna mutant egg chambers also show additional defects in nurse cell cortical actin integrity, resulting in detached cytoplasmic actin bundles and ring canals (Figure 7C). These defects closely resemble phenotypes found in *abi* mutants lacking germline Abi (pUASt-Abi rescue; Zobel and Bogdan, 2013) and *wave* germline mosaics (Zallen et al., 2002). Because overexpression of AbiΔEna in a wild-type background did not affect fertility, dominant effects can again be excluded (Figure 7D). Finally, we examined the impact of an Abi mutant lacking its Ena binding sites and the C-terminal SH3 domain (AbiΔEnaΔSH3). Consistent with our observations in the larval visual system, AbiΔEnaΔSH3 rescued the egg morphology defects and female sterility of *abi* mutants (Figures 7C–7F). In summary, our data demonstrate that the Abi:Ena interaction plays an important role in vivo in regulating diverse actin-based structures and morphogenetic processes that require a functional WRC.

DISCUSSION

Ena/VASP Proteins Cooperate with Rac during WRC-Dependent Actin Polymerization

The WRC binds and activates the Arp2/3 complex to drive actin polymerization at the plasma membrane in response to Rac signaling during cell migration (Bisi et al., 2013). In contrast, Ena/VASP proteins stimulate cell migration by antagonizing actin filament capping and acting as processive actin polymerases (Barzik et al., 2005; Bear and Gertler, 2009; Bear et al., 2002; Breitsprecher et al., 2008, 2011; Hansen and Mullins, 2010; Pasic et al., 2008). We have now demonstrated that Ena/VASP proteins can be linked to the function of WRC by virtue of a direct interaction between their EVH1 domains and Abi, an integral component of the WRC.

Our results have confirmed and extended previous yeast two-hybrid data and pull-downs from cell lysates demonstrating that the EVH1 domains of Mena and VASP can interact with human and mouse Abi1 (Dittrich et al., 2010; Hirao et al., 2006; Maruoka et al., 2012; Tani et al., 2003). The structure of several EVH1:FPPPP complexes reveals that the "FPPPP" motif adopts a type II polyproline helix that is coordinated by three aromatic residues present in all Ena/VASP family members (Peterson and Volkman, 2009). In contrast, the EVH1 domain interacts with an extended proline-rich binding site in human Abi1. Consistent with their ability to bind, Abi2 has an almost identical sequence whereas Abi3 has two "LPPPP" motifs in this region. In many respects, the extended nature of the Abi1 interaction resembles that of the N-WASP WH1 binding site in WIP, which also involves three regions of contact (Peterson et al., 2007). In classical EVH1 interactions, the acidic residues flanking the "FPPPP" motif play an important role in determining the affinity, orientation and specificity of EVH1 binding (Ball et al., 2000; Peterson and Volkman, 2009). In contrast, the EVH1 binding site in human Abi1 contains two pairs of aspartic acid residues flanking the central phenylalanine in the middle of the motif as well as a downstream acidic patch (DYEDEE; Figure 4A). The molecular basis of the EVH1 human Abi1 interaction, including the extended peptide orientation and role of acidic residues, must await structural determination of the complex. Nevertheless, our data clearly demonstrate that the EVH1 domain can bind additional proline rich ligands beyond "FPPPP" motifs.

Interestingly, the meander region of WAVE1 contains an "LPPPP" motif that is capable of interacting with Mena (Okada et al., 2012). The ability of Mena to bind Abi in the WRC presumably explains why it still associates with WAVE lacking its proline rich region (Okada et al., 2012). Consistent with the presence of "LPPPP" motifs pull-downs with recombinant proteins demonstrate that the EVH1 domain of Mena can interact with WAVE 1 and 2, but not WAVE 3 (Figure S2B). Our observations, however, suggest that the interaction with Abi is more important for Mena interactions with the WRC than WAVE (Figure 4D). Moreover, our in vitro assays clearly demonstrate that the ability of Rac to activate WRC-mediated actin polymerization via the Arp2/3 complex is significantly enhanced by VASP binding to Abi. In contrast to the full-length protein, monomeric VASP or its isolated EVH1 domain is unable to activate the WRC to stimulate Arp2/3-mediated actin polymerization even at high concentrations. This difference may reflect the ability of the VASP tetramer to induce oligomerization of the WRC, an effect that would enhance WRC potency toward the Arp2/3 complex (Padrick et al., 2008; Padrick and Rosen, 2010). It is possible that the simultaneous engagement of a VASP tetramer with Abi and the "LPPPP" motif in WAVE increases the activity of the WRC. However, oligomerization alone cannot account for our data because mutating the actin binding elements of VASP, which should have no effect on tetramerization, abrogates activity. Furthermore, the VASP effect does not appear to be simple allosteric activation of the WRC (i.e., release of the VCA), because this should produce activity equal to that of the VCA alone. While not definitive, our collective data are most consistent with a model in which VASP binds the Rac-activated WRC with high affinity based on tetramerization-mediated avidity and also interacts with actin filaments, thus

Figure 3. VASP Enhances WRC Activity in the Presence of Rac1 In Vitro
(A) VASP, but not WRC, promotes actin polymerization in the absence of Rac1.
(B) VASP further increases actin polymerization of Rac1-activated WRC, but not WRC-Abi1(1–159) or VCA.
(C) VASP increases actin polymerization of Rac1-activated WRC in a dose-dependent manner.
(D) In contrast to VASP, VASP (1–335) or EVH1 domain does not enhance the activity of Rac1-activated WRC.
(E) VASP lacking the FAB motif fails to enhance Rac1-WRC activity.
(F) VASP with a disrupted GAB motif only enhances Rac1-WRC activity at high concentrations.

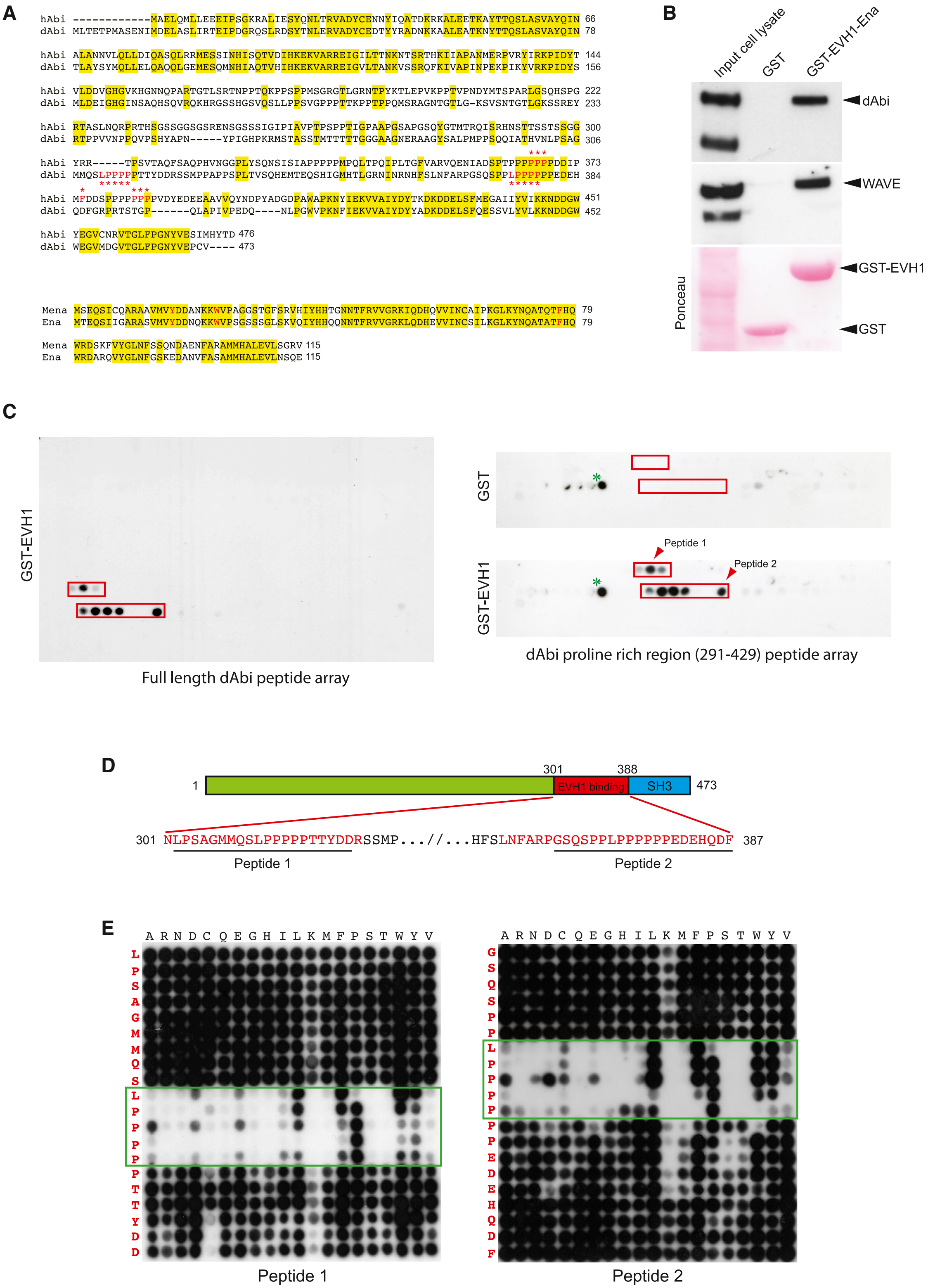

Figure 4. *Drosophila* Abi Contains Two Ena EVH1 Binding Sites

(A) Sequence alignments of human Abi (hAbi) and Mena EVH1 with their *Drosophila* counterparts, dAbi and Ena. Conserved residues are highlighted in yellow. The positions of the Abi EVH1 binding motifs and the aromatic residues responsible for binding FPPPP ligands are indicated in red.

(B) Immunoblot analysis demonstrates that the Ena EVH1 domain retains dAbi and WAVE from S2 cell extracts. The ponceau stain shows the protein resins.

(legend continued on next page)

increasing the association of the WRC with filaments. Because both the released WAVE VCA and actin filaments activate the Arp2/3 complex (Machesky et al., 1999), assembling these two elements should enhance their cooperative actions and increase actin assembly.

The Interaction of Ena/VASP with the WRC Is Conserved in Flies and Humans

In contrast to the situation in humans, the interaction between the EVH1 domain of Ena and Abi in *Drosophila* is mediated by two “LPPPP” motifs located in a proline rich region of Abi. The loss of these two “LPPPP” motifs increases the dynamics of the WRC at the plasma membrane but did not affect lamellipodium formation in S2 cells in culture. In contrast, the consequences of disrupting the interaction of Ena with Abi in vivo are more dramatic, as primary macrophages expressing Abi-ΔEna have reduced lamellipodial membrane protrusions and defects in cell morphology. Unlike the situation in S2 cells, which have been treated with dsRNA and transiently transfected with GFP-tagged expression constructs, the *abi* transgenes (Abi and AbiΔEna) are expressed from the same genomic locus. These in vivo rescue experiments therefore allow for a more precise analysis of the requirement of the interaction between Ena and Abi rather than in the hypomorphic situation in S2 cells. The ability of AbiΔEna to rescue lamellipodium formation in S2 cells might reflect an incomplete *abi* knockdown or a difference in its expression level compared to endogenous Abi in untreated cells. Consistent with this, in macrophages, we found that strong expression of Abi in earlier larval stages using the da-Gal4 driver results in a more robust rescue of lamellipodia protrusion and cell morphology defects as compared to macrophage-specific expression (hmlΔ-gal4) at late larval stages. Given that our in vitro actin polymerization assays indicate that VASP (Ena) is not an essential activator but rather acts cooperatively with Rac1 to promote WRC activation, it is likely that in vivo the requirement for this interaction depends on the level of Abi. This explanation may also partially account for the more dramatic phenotypes observed in the multicellular context.

Ena and WRC Function Together in a Complex Antagonistic Network In Vivo

Remarkably, we found that the loss of the ability of Abi to interact with Ena resulted in a similar defect in R-cell targeting as the absence of the complete protein. This suggests that Ena has a nonautonomous role in the larval brain, as we have previously shown for WRC function in targeting of early retinal axons (Stephan et al., 2011). Mosaic mutant analysis further supports a nonautonomous function for Ena in retinal axon targeting (data not shown). Thus, we propose that the interaction between Ena and the WRC is required to regulate actin dynamics in the target area neurons. However, since the precise projection pattern of early retinal axons depends on complex interactions between different populations of glia cells and neurons in the target field, it remains unclear how Ena and the WRC function together in this developmental context. In contrast, *Drosophila* oogenesis provides an excellent model to study the cell autonomous function of the interaction between Ena and the WRC.

Previous phenotypic analyses of mutant egg chambers suggest Ena and WRC have both distinct and overlapping functions during oogenesis (Gates et al., 2009; Zallen et al., 2002). Both are required for the integrity of the cortical actin in nurse cells and mutant egg chambers become multinucleated as the plasma membrane breaks down due to a loss of cortical actin integrity. In contrast, to *wave* mutant egg chambers, disruption of *ena* function does not affect ring canal morphology but rather leads to a reduced and delayed formation of cytoplasmic actin filament bundles (Gates et al., 2009; Zallen et al., 2002). Similar to *wave* germline clones, the loss of *abi* in the germline results in a dumpless mutant phenotype and female flies are sterile (Zobel and Bogdan, 2013). We have now found that these defects in egg morphology and female fertility cannot be rescued by reexpression of a full-length Abi deficient in Ena binding. AbiΔEna mutant egg chambers have defects in the integrity of the nurse cell cortical actin resulting in detached cytoplasmic actin bundles and ring canals. The rupture of nurse cell membranes is even more obvious at later stages when the fast transport of nurse cell contents starts, as recently observed for *ena*, *wave*, and *abi* mutants (Gates et al., 2009; Zallen et al., 2002; Zobel and Bogdan, 2013).

In addition to nurse cell dumping defects, we also observed a striking egg chamber elongation defect. Mutant eggs lacking the interaction between Abi and Ena fail to elongate and remain spherical as similarly found in *rac* or *pak* mutants (Conder et al., 2007). The round egg phenotype observed in flies expressing AbiΔEna suggests that there might be a defect in the basal actin cytoskeleton of the follicle cells that drives egg chamber elongation (Bilder and Haigo, 2012; Gates, 2012). Consistently, reexpression of AbiΔEna in somatic follicle cells (*abi*, *da* > UASt-AbiΔEna) also results in a round-egg phenotype. These data suggest a requirement of WRC function in follicle cells during egg elongation. Supporting this notion, we found that a follicle cell-specific knockdown of Sra-1 function results in a strong round-egg phenotype.

Our rescue experiments additionally imply a more complex interaction network among Ena, Abi, and SH3 interacting proteins. Whereas a minimal Abi fragment lacking the Ena-binding or proline-rich region and the C-terminal SH3 domain is able to rescue substantially *abi* mutant traits in *Drosophila* and *Dictyostelium* (Davidson et al., 2013; Stephan et al., 2011), the disruption of Ena-binding alone completely abolishes Abi activity. Thus, we propose a scenario in which the influence of Ena on WRC activity depends on additional proteins interacting with the Abi-SH3 domain. The most prominent candidate is the nonreceptor tyrosine kinase Abelson (Abl) that binds Abi and Ena proteins (Dai and Pendergast, 1995; Gertler et al., 1989). Based on the antagonistic genetic interaction between *ena* and *abl*, it has been

(C) Far western analysis of a dAbi peptide array reveals GST-EVH1-Ena interacts with peptides covering residues 301–322 and 362–387 in the proline-rich region of dAbi (red boxes on left). Right: GST-EVH1-Ena, but not GST, detects the same peptides. The green asterisk indicates nonspecific GST binding peptides and the red arrowheads indicate peptides selected for systematic mutagenesis.
(D) Schematic representation of dAbi and the positions of peptide 1 and 2 in the EVH1 binding peptides (red).
(E) Far western analysis reveals that mutation of the LPPPP motifs in peptide 1 or 2 has a dramatic impact on their ability to bind the EVH1 domain of Ena.

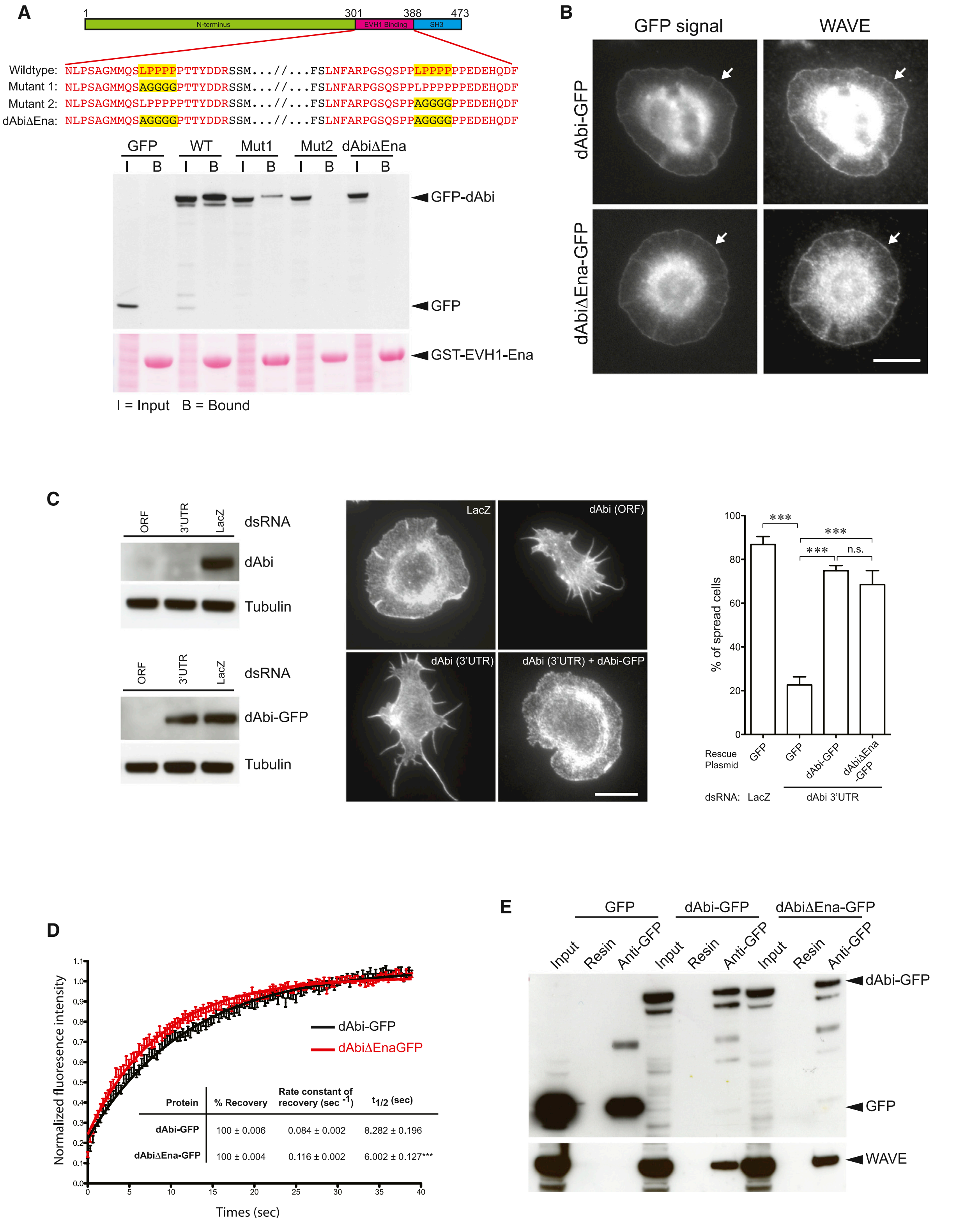

Figure 5. Ena Stabilizes dAbi at Leading Edge but Is Not Required for S2 Spreading

(A) Schematic representation of dAbi and LPPPP motif amino acid substitutions used to generate dAbiΔEna that is deficient in binding Ena. Immunoblot analysis of GST-EVH1 Ena pull-downs reveals that mutation of the two LPPPP motifs results in loss of dAbi binding. The ponceau stain shows the input cell lysates and the GST-EVH1-Ena resin.

(B) GFP-tagged dAbi and dAbiΔEna colocalize with endogenous WAVE at the plasma membrane of S2 cells (arrowheads). Scale bar represents 10 μm.

(legend continued on next page)

hypothesized that a precise balance between Abl and Ena activity is required for fly viability. However, it is still unclear how Abl affects the function of Ena, because mutation of all known Abl phosphorylation sites only has a modest effect on Ena function in vivo (Comer et al., 1998; Gertler et al., 1995; Wills et al., 1999). Similarly, Abl and Abi have opposing roles in *Drosophila* (Lin et al., 2009). Thus, we propose a model in which Ena synergizes with Rac to activate the WRC, but also inhibits Abl function. Abl in turn inhibits WRC function as previously shown (Lin et al., 2009). Thus, the disruption of Ena binding to dAbi would simultaneously decrease WRC stimulation by Ena and increase its inhibition by Abl. Such a scenario would explain why loss of Ena binding to Abi (WRC) phenocopies the *abi* mutants. This also suggests that the interaction among WRC, Abl, and Ena function is of more general relevance for actin-based processes in multicellular contexts. Furthermore, recent data also suggest that lamellipodin, which cooperates with the WRC to promote cell migration in vivo, is also likely to be part of this complex regulatory network, because it can bind both the EVH1 domain of VASP and the SH3 domain of Abi (Krause et al., 2004; Law et al., 2013).

In summary, our in vitro data clearly demonstrate that Ena/VASP proteins can directly affect the activity of the WAVE complex, whereas our observations in *Drosophila* have revealed that, in vivo, the function and activity of Ena/VASP proteins and the WAVE complex are intimately linked.

EXPERIMENTAL PROCEDURES

Escherichia coli Expression Vectors and Protein Expression

Bacterial expression vectors for GST-tagged EVH1 domains of VASP, Evl, and Mena as well as the LIM3 domain of Tes and FPPPP region of zyxin have been described (Boëda et al., 2007). The EVH1 domain of *Drosophila* Ena (residues 1–115) as well as human Abi1-3, WAVE1-3, VASP, Nap1, and PIR121 were amplified by PCR and cloned into the Not1-EcoR1 sites of pMW172-GST-3C or pMW172-HIS-3C to generate a GST or His-tagged *E. coli* expression vectors. The VASP 1–335 and the GAB (L226A/I230A/L235A/R236E/K237E) and FAB (R273E/R274E/R275E/K276E) mutants (Breitsprecher et al., 2011; Hansen and Mullins, 2010) were generated by quikchange mutagenesis. Proteins were typically expressed in BL21(DE3) Rosetta cells and purified as previously described (Boëda et al., 2007, 2011). All His-tagged VASP proteins were expressed in BL21 (DE3) $T1^R$ cells at 18°C and purified by Ni-NTA agarose beads, a Source SP15 column, and finally a Superdex200 or Superfex75 column. Other proteins, including Arp2/3 complex, actin, VCA, Rac1 Q61L, and Tev protease were purified as previously described (Ismail et al., 2009).

EVH1 Pull-Down Assays and Mass Spectrometry Analysis

Pull-down assays with recombinant proteins on MV^{D7} and S2 cell extracts were performed as previously described (Boëda et al., 2007, 2011). Stained protein bands (Imperial Protein Stain, Thermo Scientific) were reduced, alkylated, and digested with trypsin, as previously described (Collazos et al., 2011). The digests were analyzed with liquid chromatography-tandem mass spectroscopy (LC-MS/MS) on an Agilent 6510 mass spectrometer (Agilent). LC-MS/MS data were searched against a protein database (NCBInr 20080210) using the Mascot search engine (Matrix Science). All data were interrogated manually.

WRC Purification and Pull-Downs

The human WRC was purified as previously described (Chen et al., 2014). GST pull-down was performed by mixing 380 pmol of bait proteins (GST or GST-tagged EVH1 or Rac1) with equimolar prey proteins (WRC or WRC-Abi1[1–159]) and 20 μl of glutathione sepharose beads (GE Healthcare) in 1 ml of binding buffer (20 mM HEPES, 100 mM NaCl, 5% [w/v] glycerol, 2 mM $MgCl_2$, 1 mM EGTA, and 5 mM β-mercaptoethanol, pH 7) at 4°C for 30 min. Beads were centrifuged and washed three times with binding buffer. Bound proteins were eluted with 30 mM reduced glutathione and examined with SDS-PAGE.

Actin Polymerization Assays

Actin polymerization assays were performed at 22°C using a PTI Fluorometer (Photon Technology International) as previously described with slight modifications (Ismail et al., 2009). Reactions contained 4 μM rabbit muscle actin with 5% labeled with pyrene, 10 nM bovine Arp2/3 complex, 100 nM human WRC, and/or other proteins of interest in 10 mM imidazole, 100 mM KCl, 1 mM $MgCl_2$, 1 mM EGTA, 20% (w/v) glycerol, and 1 mM dithiothreitol, pH 7.0. Note that under these conditions, the WRC fully activated by Rac1 (or equivalently, 100 nM VCA) only exhibits modest activity toward the Arp2/3 complex.

Antibodies, Immunofluorescence, and Immunoblot Analysis

Monoclonal antibodies GFP 3E1 (Cancer Research UK), Actin AC-74 (Sigma), and zyxin 164D4 (SySy) as well as rabbit antibodies against Abi1 (Sigma A5106) and VASP (Cell Signaling 3132S) were used. Antibodies against human Abi1, Nap1, and PIR121 (Steffen et al., 2004) were kindly provided by Dr Theresia Stradal (University of Muenster, Germany). Antibodies against *Drosophila* Abi and WAVE have been described (Bogdan et al., 2005; Stephan et al., 2011). Immunofluorescence and immunoblot analyses were performed as described (Boëda et al., 2007). Projection patterns of all photoreceptor axons of the indicated genotypes were depicted using mouse anti-24B10 (α-Chaoptin, DSHB; Stephan et al., 2011).

HT1080 Stable Cell Lines and Scratch Assays

Human Abi1 or Abi1ΔEVH1 were cloned into the Not1/EcoRI sites of a modified pLVX-puro-GFP vector and used to generate lentiviruses. HT1080 cell lines stably expressing GFP or the GFP-tagged proteins were selected with puromycin and FACS sorting. For scratch assays, the cell lines were transfected with QIAGEN Allstars negative control or siRNA HS ABI1 9 which targets the 3′UTR of Abi1. A day later, 5×10^4 cells were seeded into each well of a 96-well ImageLock microplates (six replicate wells/condition; Essen Bioscience) and after 24 hr, a scratch was made in each well using the Essen Instruments Woundmaker 96. The cells were washed three times with PBS and serum-free Dulbecco's modified Eagle's medium was added. The plates were equilibrated at 37°C for 30 min before scanning in the Essen Instruments Incucyte FLR. Images were captured every 30 min for 20 hr and percentage relative wound density was calculated using Incucyte software. A representative graph and images from one experiment are shown. Numbers are mean of six wells ± SEM and similar results were obtained in five other experiments.

Transfections, Coimmunoprecipitation, and Far Western Analysis

All Abi mutants were generated using the Quikchange Site-directed mutagenesis Kit (Stratagene). Abi and EVH1 binding mutants were cloned into

(C) Left: The top immunoblot shows the loss of endogenous dAbi in S2 cells treated with dsRNA derived from the open reading frame (ORF) or 3′ untranslated regions (3′UTR) of the *Drosophila* Abi gene but not LacZ control. The bottom immunoblot shows the expression of GFP-tagged dAbi in S2 cells treated with the indicated dsRNA. Middle: Immunofluorescence images of S2 cells treated with dsRNA targeting LacZ (negative control) or endogenous dAbi (ORF or 3′UTR) with or without expression of dAbi-GFP. Left: Quantification of cell spreading reveals that GFP-tagged dAbi and dAbiΔEna are equally effective at rescuing the consequences of 3′UTR dsRNA-mediated loss of endogenous dAbi expression. Error bars indicate the SEM and scale bar represents 10 μm.
(D) The recovery kinetics of GFP-tagged dAbi and dAbiΔEna at the plasma membrane after photobleaching in S2 cells treated with 3′UTR dsRNA. Error bars represent SEM and n = 30. The table shows values for the percentage, rate constant, and half-time of recovery for dAbi and dAbiΔEna together with the SEM.
(E) Immunoblot analysis of immunoprecipitations reveals that GFP-tagged dAbi and dAbiΔEna incorporate into the WAVE complex. The input represents 4% of the cell lysate used in each immunoprecipitant.

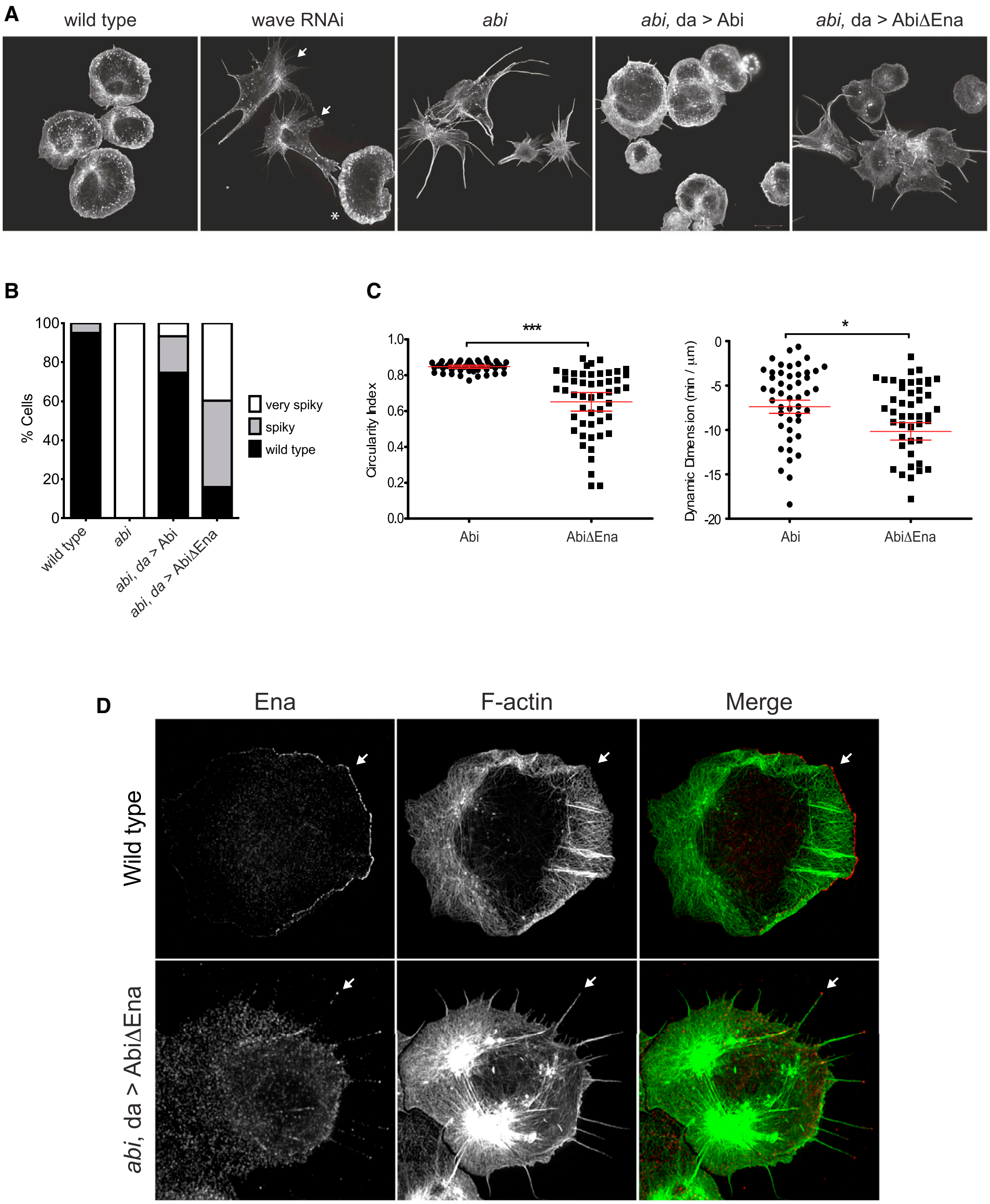

Figure 6. Ena and WRC Interactions Regulate Cell Protrusion and Dynamics

(A) Maximum intensity projection SIM images of the actin cytoskeleton in larval macrophages. Embryo-derived macrophages that do not coexpress *wave* dsRNA and GFP are indicated by the asterisk.

(B) The graph shows the frequency of cell morphology defects. N = 1,000 for each genotype and the cells were obtained from at least two independent crosses.

(C) Quantification of cell circularity and membrane protrusion rates of living larval *abi* mutant macrophages expressing Abi (n = 50) or AbiΔEna (n = 51). Error bars represent the SEM.

(D) Localization of endogenous Ena in wild-type and *abi* mutant macrophages expressing AbiΔEna. The organization of the actin cytoskeleton is severely compromised in the absence of Ena-Abi interactions.

CB6-N-GFP and pAC-C-GFP (Ac5 promoter) modified from pAc5.1/V5-His (Invitrogen) for expression in mammalian and *Drosophila* cells, respectively. 293T and S2 cells were transfected with Abi expression vectors using calcium phosphate or Effectene (QIAGEN). Coimmunoprecipitations and pull-down assays using GST-EVH1-Ena were performed as described previously (Boëda et al., 2007, 2011). Far western analysis of overlapping 20-mer Human and

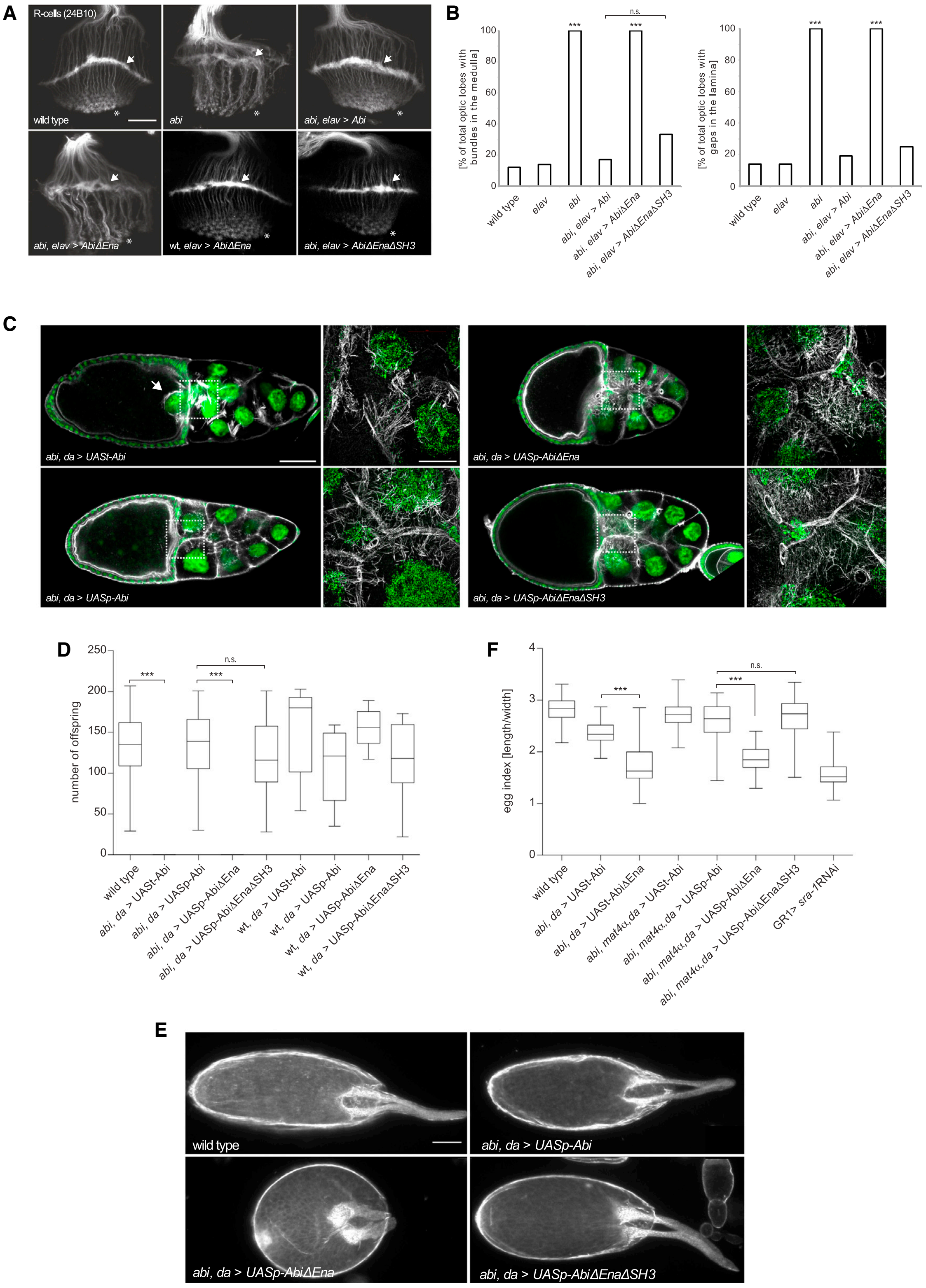

Figure 7. Ena:WRC Interactions Are Required during *Drosophila* Development

(A) Representative images of projection patterns of all photoreceptor axons of the indicated genotypes. In wild-type, R-cell axons show a stereotyped projection into the lamina (arrows) and medulla (asterisk). Loss of *abi* results in highly abnormal targeting and axonal bundling. Reexpression of Abi, but not AbiΔEna, rescues targeting defects. Scale bar represents 15 μm.

(legend continued on next page)

Drosophila Abi peptide arrays were probed with GST or GST-tagged EVH1 domains of Ena, Evl, Mena, and VASP as described previously (Postigo et al., 2006).

dsRNA Knockdown of dAbi in S2 Cells and FRAP Analysis

The 3′UTR and ORF of dAbi were amplified from a *Drosophila* cDNA library by PCR using primers containing a T7 RNA polymerase binding site at their 5′ ends. Purified PCR products served as templates for single-stranded RNA (ssRNA) synthesis using Ambion MEGAscript High yield Transcription T7 kit. The ssRNAs were annealed and the resulting double-stranded RNA (dsRNA) was purified using the Ambion MEGAclearTM kit. S2 cells were treated with dsRNA as described previously (Kiger et al., 2003). After 3 days, dAbi expression was assessed with qRT-PCR and immunoblot. To express dAbi-GFP clones, S2 cells were treated with dsRNA for 1 day before transfection. After 2 more days, cells were analyzed.

For FRAP assays, *Drosophila* S2 cells were seeded on to concanavalin A-coated MatTek dishes for 30 min and imaged using a Zeiss LSM 710 confocal with a 63×/1.4NA objective. A region of 300 × 100 pixels was recorded using a scan speed of 1.27 μs/pixel with a pinhole of 90 μm. A selected region within the imaging area of 90 × 25 pixels covering the protruding lamellipodia was imaged 5 times before bleaching with 30 iterations of the 488 nm laser at full power. Immediately after bleaching, 260 images were acquired. Analysis of fluorescence recovery and curve fitting was performed as previously described (Weisswange et al., 2009).

Fly Genetics and Microscopy

All *Drosophila* strains and crosses were performed at 25°C. The following strains were used: FRT82B *abi*Δ20 (Stephan et al., 2011), *elav*C155Gal4, da-gal4, *hml*Δ-gal4, and EGFP (Bloomington Stock Center). UAS-*wave*dsRNA transgenic flies were obtained from the Japanese National Institute of Genetics. UASt/p-Abi, UASt/p-AbiΔEna and UASt/p-AbiΔEnaΔSH3 transgenes were generated by ΦC31-integrase-mediated integration into the landing site M{3xP3-RFP.attP'}ZH-68E as previously described (Stephan et al., 2011). Full-length Abi, AbiΔEna, and AbiΔEnaΔSH3 fragments were amplified by PCR and cloned into Gateway Entry Vectors (pENTR DTOPO, Invitrogen). The inserts were sequenced and subcloned into pUASt-attB-rfA, pUASp-attB-rfA pUASt-attB-rfA-EGFP (*Drosophila* Genomics Resource Center) by in vitro lambda recombination (Invitrogen). To force early expression and to simultaneously depict cell dynamics, we combined the ubiquitous *da*-gal4 driver with the macrophage-specific expression of cytoplasmic EGFP (*hml*Δ-gal4, UAS-EGFP). Macrophages were isolated by dissecting larvae in a drop of M3 medium (Invitrogen). Cells from 10 larvae were plated on a glass slide for 60 min. Glass slides were pretreated with 0.5 mg/ml ConcanavalinA (Sigma) for 30 min. Cells were fixed for 15 min in 4% paraformaldehyde, and shortly rinsed in PBS and 0.1% Triton (PBST). After fixation, cells were incubated for 2 hr with primary antibody, rinsed twice with PBS, and incubated for 1 hr with secondary antibody and with phalloidin-Alexa488 (1:100) and DAPI (1:1000, Invitrogen).

Wild-type ovaries (w^{1118}) and ovaries from rescued abiΔ20 mutant females (abiΔ20, da-gal4/abiΔ20, UASp-Abi; abiΔ20, da-gal4/abiΔ20, UASp-AbiΔEna and abiΔ20, da-gal4/abiΔ20, UASp-AbiΔEnaΔSH3) were dissected in cold PBS, fixed in 4% paraformaldehyde in PBS for 20 min, and stained with Alexa-Fluor-*488* phalloidin and DAPI (Invitrogen). To test fertility, one mutant female was mated with one wild-type male. The number of offspring was counted after 15 days. SIM images were taken with an ELYRA S.1 Microscope (Zeiss) with the software Zen 2010 D (Zeiss). For image acquisition, five grid rotations were used with an average of two.

For live imaging, cells were plated on chambered cover glass (Lab-Tek) without concanvalinA. Movies were taken with a spinning disc Cell Observer SD Zeiss microscope and cropped so that only a single cell was present within the field of view. Following Kalman-filtering to suppress noise, cells were segmented in each frame using a region-growing algorithm implemented as a plug-in for ImageJ and velocity maps constructed as described previously (Döbereiner et al., 2006). The circularity of cells ($C = 4\pi[\text{area}]/[\text{perimeter}]^2$) was estimated in each movie frame and an average taken for all frames. The dynamic dimension is a measure we define as the slope of the line of best fit to the histogram of a velocity map, plotted logarithmically, for velocities greater than or equal to zero ($R2 > 0.9$). The software necessary to perform these calculations was coded in Java and implemented as a plug-in for ImageJ.

SUPPLEMENTAL INFORMATION

Supplemental Information includes three figures and one movie and can be found with this article online at http://dx.doi.org/10.1016/j.devcel.2014.08.001.

AUTHOR CONTRIBUTIONS

The project was conceived by M.W. X.J.C. identified and mapped all Ena/VASP EVH1 interactions in human and *Drosophila* Abi. X.J.C also generated all Abi mutants and VASP 1-335 and performed all experiments in S2 cells, including the FRAP analysis. X.J.C. was guided by M.C.M. and M.W. A.J.S., R.S., and B.C. contributed equally to this work. A.J.S. and R.S. performed and analyzed the fly fertility/oogenesis and photoreceptor experiments, respectively. S.B. oversaw all experimentation on flies and performed experiments on fly hemocytes. B.C. purified the WRC and VASP proteins and performed GST-EVH1/WRC pull downs, as well as all in vitro actin polymerization assays, which were overseen by M.K.R. T.E.H. generated HT1080 cell lines, analyzed their migration, generated VASP GAB and FAB mutants, and performed Abi1-3 and WAVE1-3 in vitro EVH1 pull-down assays. D.J.B performed image analysis of fly macrophages. M.K.R., S.B., and M.W. wrote the paper with assistance from all authors.

ACKNOWLEDGMENTS

We thank Theresia Stradal (Helmholtz Centre for Infection Research) and Frank Gertler (MIT) for providing antibodies against human WAVE components and MV^{D7} cells, respectively. We also thank Laura Machesky (Beatson Institute), Theresia Stradal, and Shiro Suetsugu (Nara Institute of Science and Technology) for WAVE1, WAVE2, and WAVE3 clones, respectively. We also thank the London Research Institute Protein Analysis & Proteomics, Peptide Synthesis, Light Microscopy services, Nic Tapon, and Barry Thompson laboratories for technical support and reagents, as well as members of the Way laboratory, Yanxiang Zhou, and Helen Walden for comments on the manuscript. This work was supported by Cancer Research UK (X.J.C., D.J.B., M.C.M., and M.W.); the Deutsche Forschungsgemeinschaft (DFG) and the Cells-in-Motion Cluster of Excellence (EXC 1003 – CiM), University of Münster (A.J.S., R.S., and S.B.); the Howard Hughes Medical Institute; and grants from the NIH (R01-GM56322) and Welch Foundation (I–1544; to B.C. and M.K.R.).

(B) Quantification of the frequency of optic lobes with axonal bundles and the number of axonal bundles in the medulla per optic lobe of the indicated genotypes. ***$p < 0.001$ (ANOVA). Number of optic lobes analyzed: wild-type = 38; abi = 36; Abi = 37; AbiΔEna = 30.
(C) Confocal images of the actin cytoskeleton (white) and nuclei (green) of stage 10B egg chambers of the indicated genotypes. Arrow marks nuclei entering the oocyte and scale bar represents 50 μm. The boxes correspond to the structured illumination microscopy images in which the scale bar represents 20 μm.
(D) Quantification of female fertility for the different genotypes. Box plots depicting the number of offspring counted from one female mated to one wild-type male. (Rescued genotypes n = 24 and gain-of-function genotypes n = 10.) ***$p < 0.001$ (ANOVA). There are no significant differences (n.s.) between overexpression of Abi, AbiΔEna, and AbiΔEnaΔSH3.
(E) Bright-field micrographs of *Drosophila* eggs of indicated genotypes. Scale bars represent 150 μm.
(F) Box plot shows the egg index that represents the quotient of length divided by width. It indicates the relative egg size of the indicated genotypes (n = 100). ***$p < 0.001$ (ANOVA).

Received: November 20, 2013
Revised: July 21, 2014
Accepted: August 1, 2014
Published: September 8, 2014

REFERENCES

Ball, L.J., Kühne, R., Hoffmann, B., Häfner, A., Schmieder, P., Volkmer-Engert, R., Hof, M., Wahl, M., Schneider-Mergener, J., Walter, U., et al. (2000). Dual epitope recognition by the VASP EVH1 domain modulates polyproline ligand specificity and binding affinity. EMBO J. *19*, 4903–4914.

Barzik, M., Kotova, T.I., Higgs, H.N., Hazelwood, L., Hanein, D., Gertler, F.B., and Schafer, D.A. (2005). Ena/VASP proteins enhance actin polymerization in the presence of barbed end capping proteins. J. Biol. Chem. *280*, 28653–28662.

Bear, J.E., and Gertler, F.B. (2009). Ena/VASP: towards resolving a pointed controversy at the barbed end. J. Cell Sci. *122*, 1947–1953.

Bear, J.E., Loureiro, J.J., Libova, I., Fässler, R., Wehland, J., and Gertler, F.B. (2000). Negative regulation of fibroblast motility by Ena/VASP proteins. Cell *101*, 717–728.

Bear, J.E., Svitkina, T.M., Krause, M., Schafer, D.A., Loureiro, J.J., Strasser, G.A., Maly, I.V., Chaga, O.Y., Cooper, J.A., Borisy, G.G., and Gertler, F.B. (2002). Antagonism between Ena/VASP proteins and actin filament capping regulates fibroblast motility. Cell *109*, 509–521.

Bilder, D., and Haigo, S.L. (2012). Expanding the morphogenetic repertoire: perspectives from the Drosophila egg. Dev. Cell *22*, 12–23.

Bisi, S., Disanza, A., Malinverno, C., Frittoli, E., Palamidessi, A., and Scita, G. (2013). Membrane and actin dynamics interplay at lamellipodia leading edge. Curr. Opin. Cell Biol. *25*, 565–573.

Boëda, B., Briggs, D.C., Higgins, T., Garvalov, B.K., Fadden, A.J., McDonald, N.Q., and Way, M. (2007). Tes, a specific Mena interacting partner, breaks the rules for EVH1 binding. Mol. Cell *28*, 1071–1082.

Boëda, B., Knowles, P.P., Briggs, D.C., Murray-Rust, J., Soriano, E., Garvalov, B.K., McDonald, N.Q., and Way, M. (2011). Molecular recognition of the Tes LIM2-3 domains by the actin-related protein Arp7A. J. Biol. Chem. *286*, 11543–11554.

Bogdan, S., Stephan, R., Löbke, C., Mertens, A., and Klämbt, C. (2005). Abi activates WASP to promote sensory organ development. Nat. Cell Biol. *7*, 977–984.

Breitsprecher, D., Kiesewetter, A.K., Linkner, J., Urbanke, C., Resch, G.P., Small, J.V., and Faix, J. (2008). Clustering of VASP actively drives processive, WH2 domain-mediated actin filament elongation. EMBO J. *27*, 2943–2954.

Breitsprecher, D., Kiesewetter, A.K., Linkner, J., Vinzenz, M., Stradal, T.E., Small, J.V., Curth, U., Dickinson, R.B., and Faix, J. (2011). Molecular mechanism of Ena/VASP-mediated actin-filament elongation. EMBO J. *30*, 456–467.

Chen, B., Padrick, S.B., Henry, L., and Rosen, M.K. (2014). Biochemical reconstitution of the WAVE regulatory complex. Methods Enzymol. *540*, 55–72.

Collazos, A., Michael, N., Whelan, R.D., Kelly, G., Mellor, H., Pang, L.C., Totty, N., and Parker, P.J. (2011). Site recognition and substrate screens for PKN family proteins. Biochem. J. *438*, 535–543.

Coló, G.P., Lafuente, E.M., and Teixidó, J. (2012). The MRL proteins: adapting cell adhesion, migration and growth. Eur. J. Cell Biol. *91*, 861–868.

Comer, A.R., Ahern-Djamali, S.M., Juang, J.L., Jackson, P.D., and Hoffmann, F.M. (1998). Phosphorylation of Enabled by the Drosophila Abelson tyrosine kinase regulates the in vivo function and protein-protein interactions of Enabled. Mol. Cell. Biol. *18*, 152–160.

Conder, R., Yu, H., Zahedi, B., and Harden, N. (2007). The serine/threonine kinase dPak is required for polarized assembly of F-actin bundles and apical-basal polarity in the Drosophila follicular epithelium. Dev. Biol. *305*, 470–482.

Coutts, A.S., MacKenzie, E., Griffith, E., and Black, D.M. (2003). TES is a novel focal adhesion protein with a role in cell spreading. J. Cell Sci. *116*, 897–906.

Dai, Z., and Pendergast, A.M. (1995). Abi-2, a novel SH3-containing protein interacts with the c-Abl tyrosine kinase and modulates c-Abl transforming activity. Genes Dev. *9*, 2569–2582.

Davidson, A.J., Ura, S., Thomason, P.A., Kalna, G., and Insall, R.H. (2013). Abi is required for modulation and stability but not localization or activation of the SCAR/WAVE complex. Eukaryot. Cell *12*, 1509–1516.

Dittrich, M., Strassberger, V., Fackler, M., Tas, P., Lewandrowski, U., Sickmann, A., Walter, U., Dandekar, T., and Birschmann, I. (2010). Characterization of a novel interaction between vasodilator-stimulated phosphoprotein and Abelson interactor 1 in human platelets: a concerted computational and experimental approach. Arterioscler. Thromb. Vasc. Biol. *30*, 843–850.

Döbereiner, H.G., Dubin-Thaler, B.J., Hofman, J.M., Xenias, H.S., Sims, T.N., Giannone, G., Dustin, M.L., Wiggins, C.H., and Sheetz, M.P. (2006). Lateral membrane waves constitute a universal dynamic pattern of motile cells. Phys. Rev. Lett. *97*, 038102.

Drees, B., Friederich, E., Fradelizi, J., Louvard, D., Beckerle, M.C., and Golsteyn, R.M. (2000). Characterization of the interaction between zyxin and members of the Ena/vasodilator-stimulated phosphoprotein family of proteins. J. Biol. Chem. *275*, 22503–22511.

Garvalov, B.K., Higgins, T.E., Sutherland, J.D., Zettl, M., Scaplehorn, N., Köcher, T., Piddini, E., Griffiths, G., and Way, M. (2003). The conformational state of Tes regulates its zyxin-dependent recruitment to focal adhesions. J. Cell Biol. *161*, 33–39.

Gates, J. (2012). Drosophila egg chamber elongation: insights into how tissues and organs are shaped. Fly (Austin) *6*, 213–227.

Gates, J., Nowotarski, S.H., Yin, H., Mahaffey, J.P., Bridges, T., Herrera, C., Homem, C.C., Janody, F., Montell, D.J., and Peifer, M. (2009). Enabled and Capping protein play important roles in shaping cell behavior during Drosophila oogenesis. Dev. Biol. *333*, 90–107.

Gertler, F.B., Bennett, R.L., Clark, M.J., and Hoffmann, F.M. (1989). Drosophila abl tyrosine kinase in embryonic CNS axons: a role in axonogenesis is revealed through dosage-sensitive interactions with disabled. Cell *58*, 103–113.

Gertler, F.B., Comer, A.R., Juang, J.L., Ahern, S.M., Clark, M.J., Liebl, E.C., and Hoffmann, F.M. (1995). enabled, a dosage-sensitive suppressor of mutations in the Drosophila Abl tyrosine kinase, encodes an Abl substrate with SH3 domain-binding properties. Genes Dev. *9*, 521–533.

Hansen, S.D., and Mullins, R.D. (2010). VASP is a processive actin polymerase that requires monomeric actin for barbed end association. J. Cell Biol. *191*, 571–584.

Hirao, N., Sato, S., Gotoh, T., Maruoka, M., Suzuki, J., Matsuda, S., Shishido, T., and Tani, K. (2006). NESH (Abi-3) is present in the Abi/WAVE complex but does not promote c-Abl-mediated phosphorylation. FEBS Lett. *580*, 6464–6470.

Hoffman, L.M., Jensen, C.C., Kloeker, S., Wang, C.L., Yoshigi, M., and Beckerle, M.C. (2006). Genetic ablation of zyxin causes Mena/VASP mislocalization, increased motility, and deficits in actin remodeling. J. Cell Biol. *172*, 771–782.

Hudson, A.M., and Cooley, L. (2002). A subset of dynamic actin rearrangements in Drosophila requires the Arp2/3 complex. J. Cell Biol. *156*, 677–687.

Ismail, A.M., Padrick, S.B., Chen, B., Umetani, J., and Rosen, M.K. (2009). The WAVE regulatory complex is inhibited. Nat. Struct. Mol. Biol. *16*, 561–563.

Jenzora, A., Behrendt, B., Small, J.V., Wehland, J., and Stradal, T.E. (2005). PREL1 provides a link from Ras signalling to the actin cytoskeleton via Ena/VASP proteins. FEBS Lett. *579*, 455–463.

Kiger, A.A., Baum, B., Jones, S., Jones, M.R., Coulson, A., Echeverri, C., and Perrimon, N. (2003). A functional genomic analysis of cell morphology using RNA interference. J. Biol. *2*, 27.

Krause, M., Leslie, J.D., Stewart, M., Lafuente, E.M., Valderrama, F., Jagannathan, R., Strasser, G.A., Rubinson, D.A., Liu, H., Way, M., et al. (2004). Lamellipodin, an Ena/VASP ligand, is implicated in the regulation of lamellipodial dynamics. Dev. Cell *7*, 571–583.

Kunda, P., Craig, G., Dominguez, V., and Baum, B. (2003). Abi, Sra1, and Kette control the stability and localization of SCAR/WAVE to regulate the formation of actin-based protrusions. Curr. Biol. *13*, 1867–1875.

Lafuente, E.M., van Puijenbroek, A.A., Krause, M., Carman, C.V., Freeman, G.J., Berezovskaya, A., Constantine, E., Springer, T.A., Gertler, F.B., and

Boussiotis, V.A. (2004). RIAM, an Ena/VASP and Profilin ligand, interacts with Rap1-GTP and mediates Rap1-induced adhesion. Dev. Cell *7*, 585–595.

Law, A.L., Vehlow, A., Kotini, M., Dodgson, L., Soong, D., Theveneau, E., Bodo, C., Taylor, E., Navarro, C., Perera, U., et al. (2013). Lamellipodin and the Scar/WAVE complex cooperate to promote cell migration in vivo. J. Cell Biol. *203*, 673–689.

Lin, T.Y., Huang, C.H., Kao, H.H., Liou, G.G., Yeh, S.R., Cheng, C.M., Chen, M.H., Pan, R.L., and Juang, J.L. (2009). Abi plays an opposing role to Abl in Drosophila axonogenesis and synaptogenesis. Development *136*, 3099–3107.

Machesky, L.M., Mullins, R.D., Higgs, H.N., Kaiser, D.A., Blanchoin, L., May, R.C., Hall, M.E., and Pollard, T.D. (1999). Scar, a WASp-related protein, activates nucleation of actin filaments by the Arp2/3 complex. Proc. Natl. Acad. Sci. USA *96*, 3739–3744.

Maruoka, M., Sato, M., Yuan, Y., Ichiba, M., Fujii, R., Ogawa, T., Ishida-Kitagawa, N., Takeya, T., and Watanabe, N. (2012). Abl-1-bridged tyrosine phosphorylation of VASP by Abelson kinase impairs association of VASP to focal adhesions and regulates leukaemic cell adhesion. Biochem. J. *441*, 889–899.

Niebuhr, K., Ebel, F., Frank, R., Reinhard, M., Domann, E., Carl, U.D., Walter, U., Gertler, F.B., Wehland, J., and Chakraborty, T. (1997). A novel proline-rich motif present in ActA of Listeria monocytogenes and cytoskeletal proteins is the ligand for the EVH1 domain, a protein module present in the Ena/VASP family. EMBO J. *16*, 5433–5444.

Okada, H., Uezu, A., Soderblom, E.J., Moseley, M.A., 3rd, Gertler, F.B., and Soderling, S.H. (2012). Peptide array X-linking (PAX): a new peptide-protein identification approach. PLoS ONE *7*, e37035.

Padrick, S.B., and Rosen, M.K. (2010). Physical mechanisms of signal integration by WASP family proteins. Annu. Rev. Biochem. *79*, 707–735.

Padrick, S.B., Cheng, H.C., Ismail, A.M., Panchal, S.C., Doolittle, L.K., Kim, S., Skehan, B.M., Umetani, J., Brautigam, C.A., Leong, J.M., and Rosen, M.K. (2008). Hierarchical regulation of WASP/WAVE proteins. Mol. Cell *32*, 426–438.

Pasic, L., Kotova, T., and Schafer, D.A. (2008). Ena/VASP proteins capture actin filament barbed ends. J. Biol. Chem. *283*, 9814–9819.

Peterson, F.C., and Volkman, B.F. (2009). Diversity of polyproline recognition by EVH1 domains. Front Biosci (Landmark Ed) *14*, 833–846.

Peterson, F.C., Deng, Q., Zettl, M., Prehoda, K.E., Lim, W.A., Way, M., and Volkman, B.F. (2007). Multiple WASP-interacting protein recognition motifs are required for a functional interaction with N-WASP. J. Biol. Chem. *282*, 8446–8453.

Postigo, A., Cross, J.R., Downward, J., and Way, M. (2006). Interaction of F1L with the BH3 domain of Bak is responsible for inhibiting vaccinia-induced apoptosis. Cell Death Differ. *13*, 1651–1662.

Quinn, C.C., Pfeil, D.S., Chen, E., Stovall, E.L., Harden, M.V., Gavin, M.K., Forrester, W.C., Ryder, E.F., Soto, M.C., and Wadsworth, W.G. (2006). UNC-6/netrin and SLT-1/slit guidance cues orient axon outgrowth mediated by MIG-10/RIAM/lamellipodin. Curr. Biol. *16*, 845–853.

Rogers, S.L., Wiedemann, U., Stuurman, N., and Vale, R.D. (2003). Molecular requirements for actin-based lamella formation in Drosophila S2 cells. J. Cell Biol. *162*, 1079–1088.

Rørth, P. (1998). Gal4 in the Drosophila female germline. Mech. Dev. *78*, 113–118.

Sander, M., Squarr, A.J., Risse, B., Jiang, X., and Bogdan, S. (2013). Drosophila pupal macrophages—a versatile tool for combined ex vivo and in vivo imaging of actin dynamics at high resolution. Eur. J. Cell Biol. *92*, 349–354.

Steffen, A., Rottner, K., Ehinger, J., Innocenti, M., Scita, G., Wehland, J., and Stradal, T.E. (2004). Sra-1 and Nap1 link Rac to actin assembly driving lamellipodia formation. EMBO J. *23*, 749–759.

Stephan, R., Gohl, C., Fleige, A., Klämbt, C., and Bogdan, S. (2011). Membrane-targeted WAVE mediates photoreceptor axon targeting in the absence of the WAVE complex in Drosophila. Mol. Biol. Cell *22*, 4079–4092.

Tani, K., Sato, S., Sukezane, T., Kojima, H., Hirose, H., Hanafusa, H., and Shishido, T. (2003). Abl interactor 1 promotes tyrosine 296 phosphorylation of mammalian enabled (Mena) by c-Abl kinase. J. Biol. Chem. *278*, 21685–21692.

Weisswange, I., Newsome, T.P., Schleich, S., and Way, M. (2009). The rate of N-WASP exchange limits the extent of ARP2/3-complex-dependent actin-based motility. Nature *458*, 87–91.

Wills, Z., Bateman, J., Korey, C.A., Comer, A., and Van Vactor, D. (1999). The tyrosine kinase Abl and its substrate enabled collaborate with the receptor phosphatase Dlar to control motor axon guidance. Neuron *22*, 301–312.

Zallen, J.A., Cohen, Y., Hudson, A.M., Cooley, L., Wieschaus, E., and Schejter, E.D. (2002). SCAR is a primary regulator of Arp2/3-dependent morphological events in Drosophila. J. Cell Biol. *156*, 689–701.

Zobel, T., and Bogdan, S. (2013). A high resolution view of the fly actin cytoskeleton lacking a functional WAVE complex. J. Microsc. *251*, 224–231.

Developmental Cell, Volume *30*

Supplemental Information

Ena/VASP Proteins Cooperate with the WAVE Complex to Regulate the Actin Cytoskeleton

Xing Judy Chen, Anna Julia Squarr, Raiko Stephan, Baoyu Chen, Theresa E. Higgins, David J. Barry, Morag C. Martin, Michael K. Rosen, Sven Bogdan, and Michael Way

Supplemental figure legends

Figure S1: The interaction between the EVH1 domain of Ena/VASP proteins and Abi promotes cell migration, related to figure 2. (A) Far western analysis with GST or the GST-EVH1 domains of Mena, VASP and Evl on arrays of overlapping peptides covering residues 320-415 of human Abi1. Similar peptides were found to interact with all three EVH1 domains. Red arrowheads indicate the examples of non-identical peptide binding patterns. Green arrows correspond to the first and last peptides listed in (B). **(B)** Shows a list and relative binding of human Abi1 peptides to the EVH1 domains of Mena, VASP and Evl. **(C)** Immunoblot analysis of Abi1 in HT1080 cells stably expressing the indicated GFP tagged protein treated with control (Ctrl) or siRNA against the 3' UTR of Abi1 message (Abi). Long and short exposures with anti-GFP reveal that GFP is greatly over expressed compared to GFP-Abi1. Anti-Abi1, however, reveals that the GFP-Abi1 proteins are expressed at comparable levels to endogenous Abi1 (see GFP Ctrl lane). **(D)** Phase images of HT1080 cells treated with the indicated siRNA and expressing GFP, GFP-Abi or Abi1ΔEVH1 migrating into a scratch at the indicated time. The yellow lines correspond to the migration front and the scale bar = 300µm.

Figure S2: Purified WRC and VASP proteins used for in vitro actin polymerization assays, related to figure 3. (A) Coomassie stained SDS-PAGE gel showing purified recombinant proteins used in actin polymerization assays. **(B)** In *vitro* pull-down assays with recombinant proteins reveals that the Mena EVH1 domain interacts directly with WAVE1 and WAVE2 but not WAVE3.

Figure S3: Overexpression of the Abi variants in a wild type background does not lead to dominant negative effects on axon targetting, related to figure 7.
(A) Representative images of projection patterns of all photoreceptor axons of the indicated genotypes. Scale bar = 15µm **(B)** Quantification of the frequency of optic lobes with axonal bundles and the number of gaps per optic lobe of the indicated genotypes. * indicates $p < 0.05$ (ANOVA).

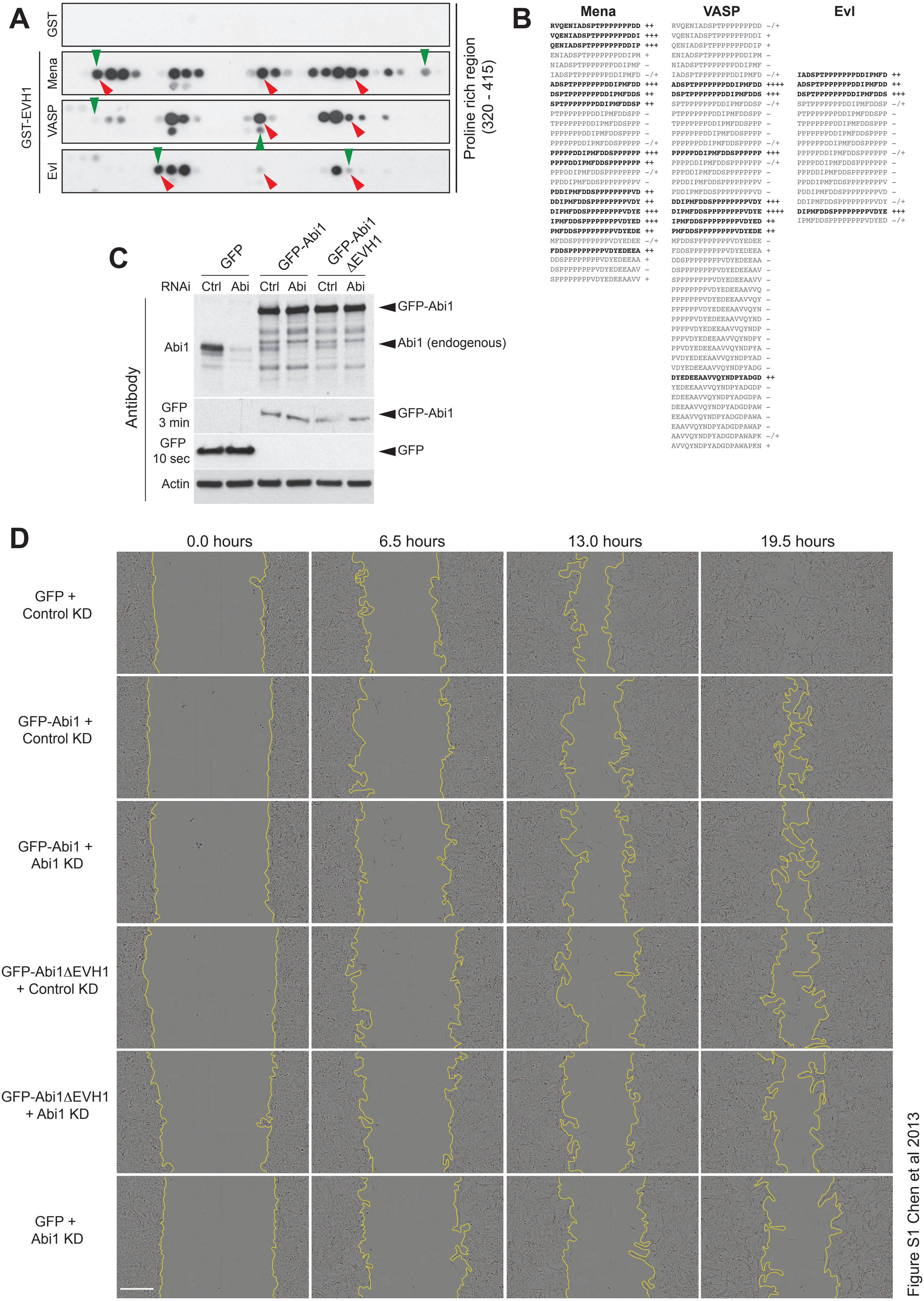

Figure S1 Chen et al 2013

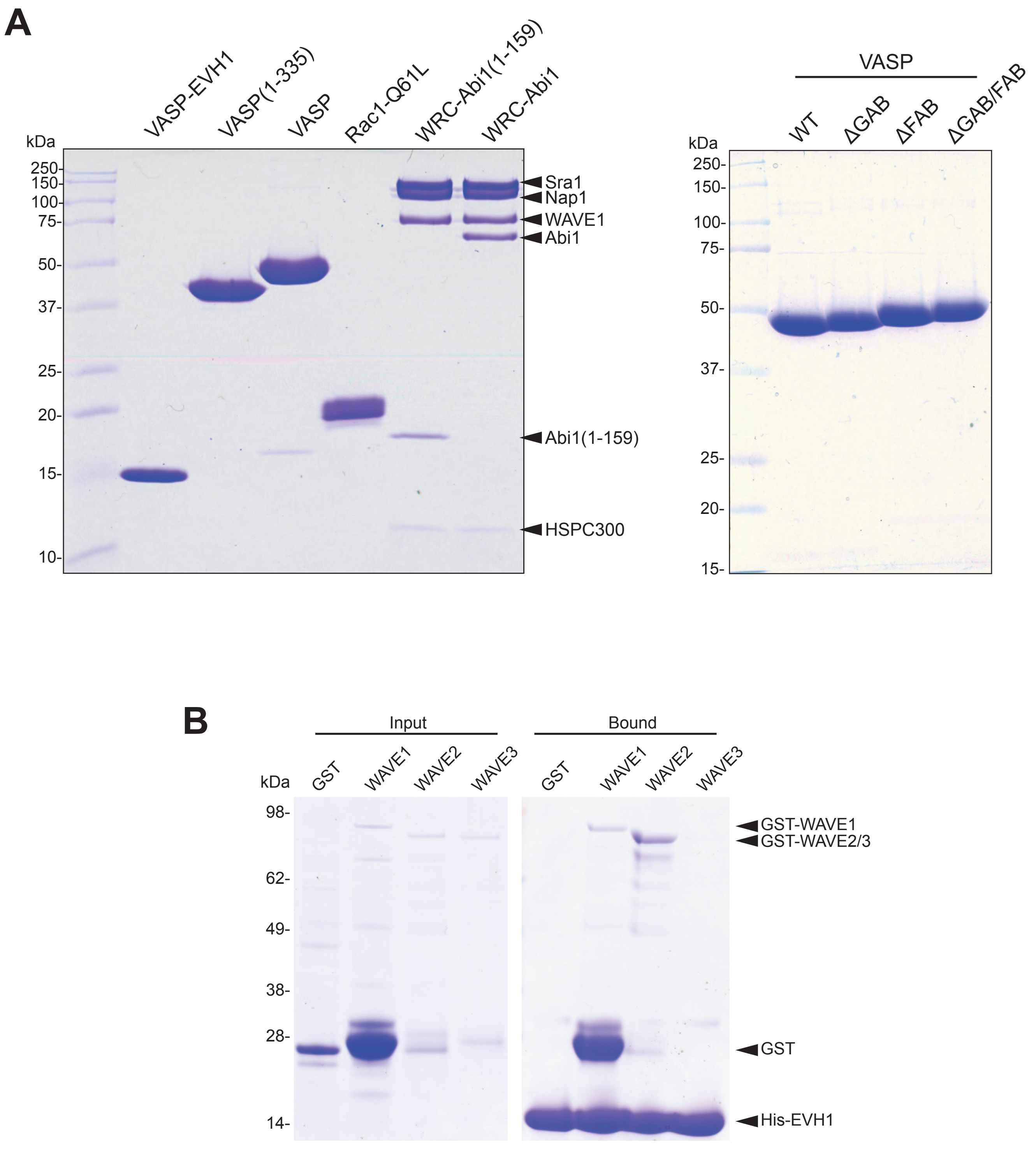

Figure S2 Chen et al 2013

A

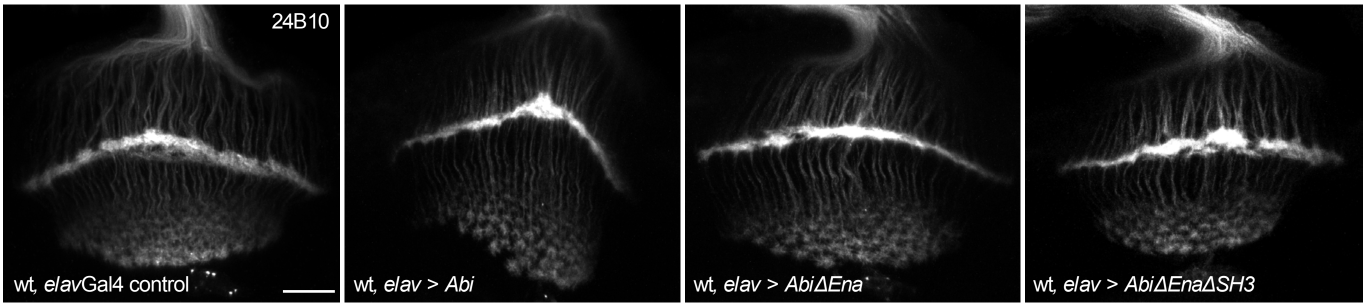

B

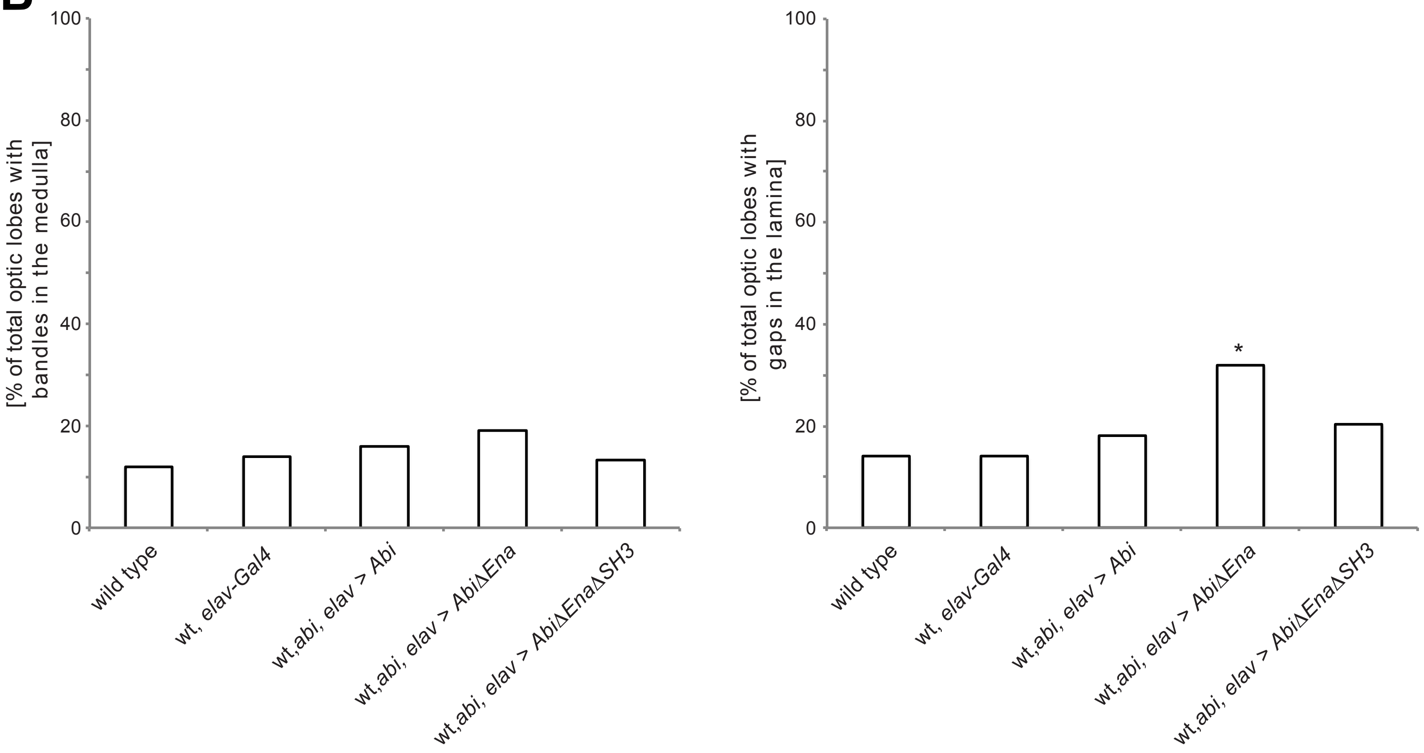

Figure S3 Chen et al 2013

Supplemental Movie legends

Movie S1: Representative movie of larval Abi mutant macrophages expressing dAbi and dAbiΔEna, related to figure 6. The segmented cell boundaries are color-coded to represent the local membrane velocity – green depicts membrane expansion and red retraction. The time is shown in minutes and seconds.
